# Supplementary figures and images for: Neurocognitive processing efficiency for discriminating human non-alarm rather than alarm scream calls
Source: PLoS Biol. 2021 Apr 13;19(4):e3000751. doi: 10.1371/journal.pbio.3000751 (PMC8043411; doi:10.1371/journal.pbio.3000751)

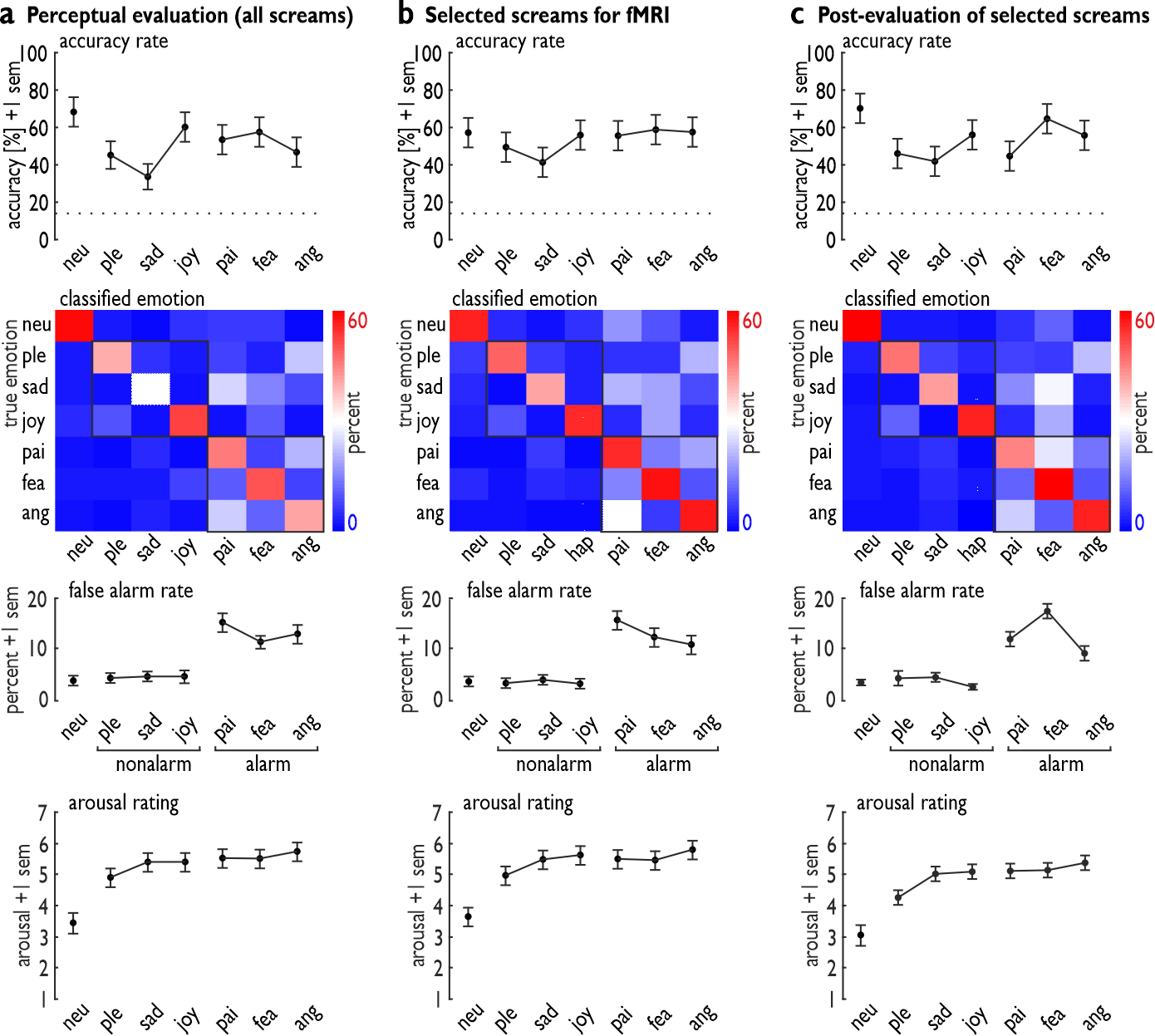

Supplement: S1 Fig — (a) Overall rating of the total n = 420 screams by n = 26 participants with respect to the recognition rate (top), their confusion matrix (middle top), the false alarm rate quantifying which categories were used during misclassifications (middle bottom), and the arousal ratings of each scream type (bottom). (b) The same measure for the selected n = 84 screams that were used in the behavioral and the fMRI experiments. (c) Participants in the fMRI experiment also performed a post-experimental rating of the same selected screams as in (b). Numerical data underlying the plots (a–c) can be found in S4 Data; see S1 Text for statistical analyses. (TIF) [file pbio.3000751.s005.tif]

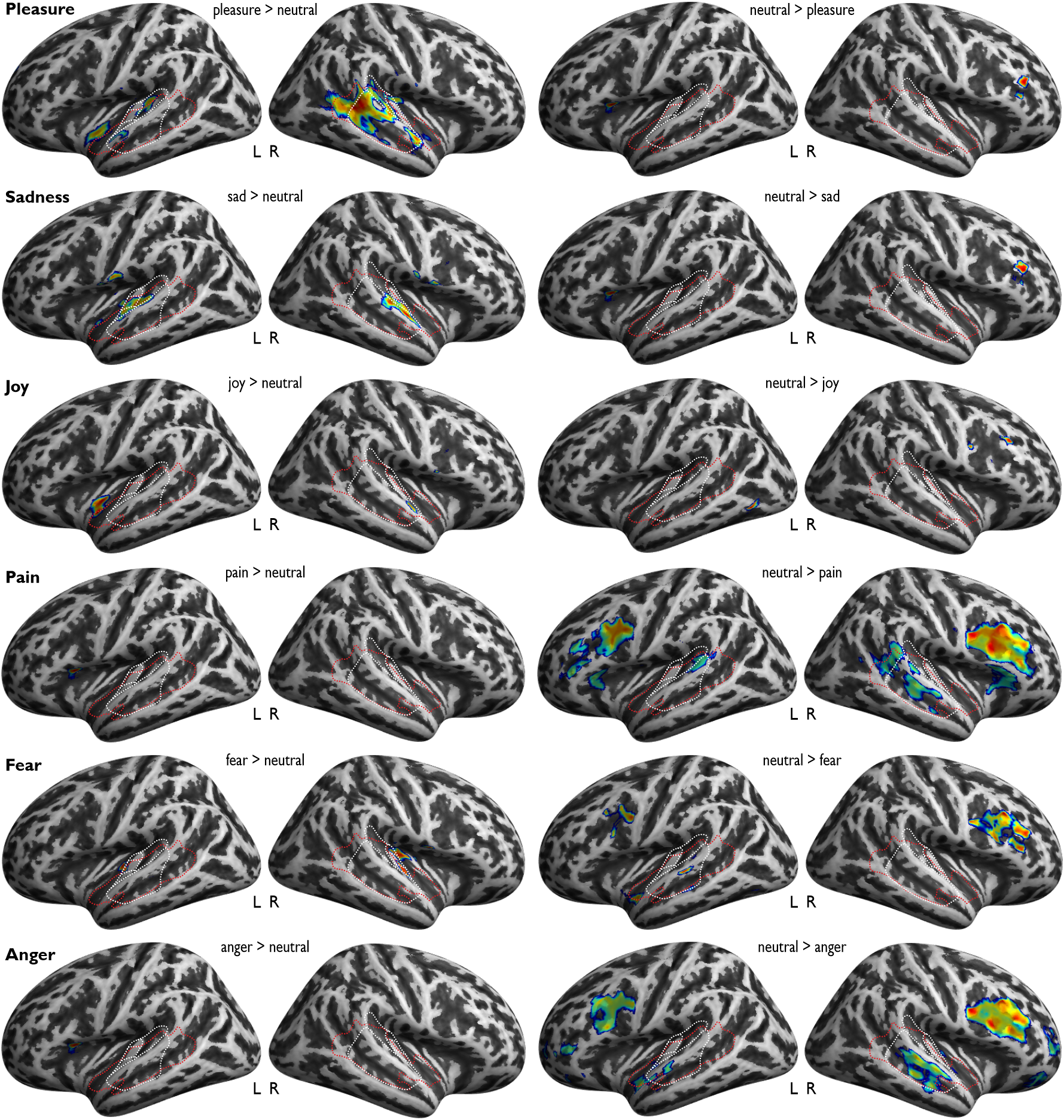

Supplement: S2 Fig — Functional activation for contrasting each of the 6 scream types against neutral screams (n = 30). This resulted in either “positive” activations (left 2 columns: higher activity compared with neutral screams) or “negative” activations (deactivations) (right 2 columns: lower activity compared with neutral screams). Threshold p = 0.005 voxel level, cluster size of k = 42 (corrected p = 0.05 at cluster level). (TIF) [file pbio.3000751.s006.tif]

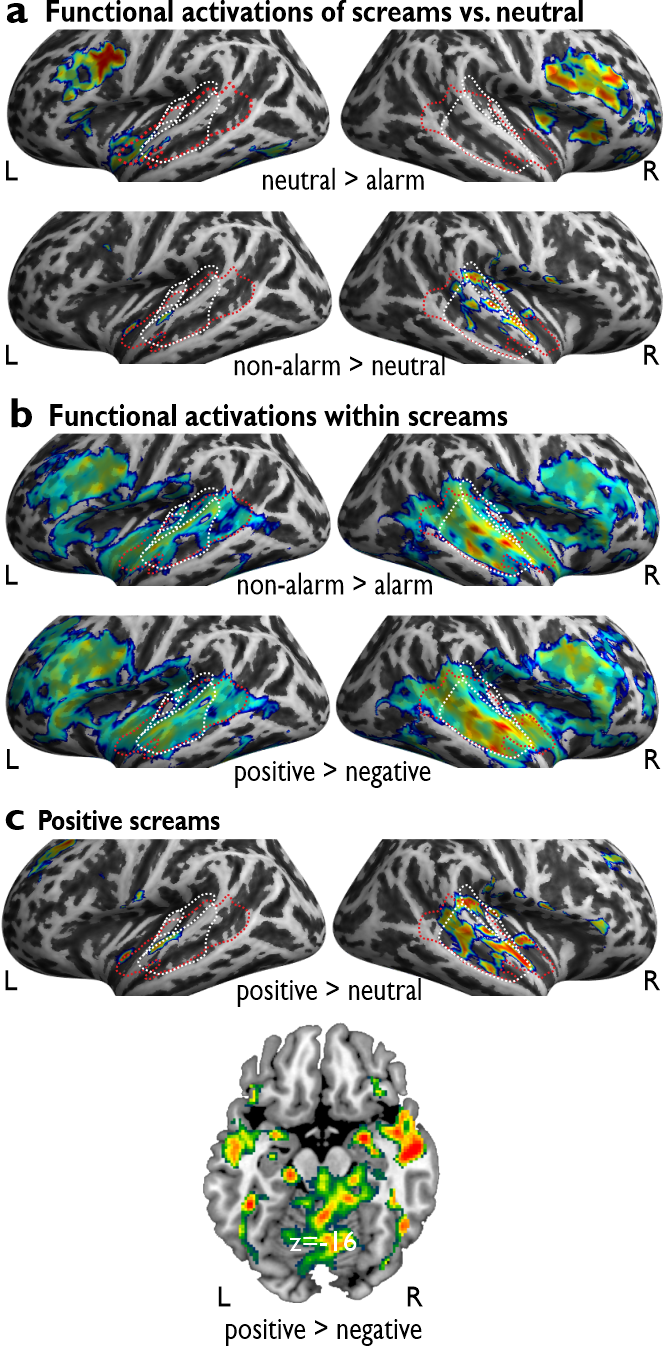

Supplement: S3 Fig — (a) Functional activations for alarm and non-alarm screams compared with neutral screams, with trials sorted on the basis of how participants (n = 30) classified each scream in the post-experimental rating. The analysis included 7 types of screams similar to those in the original analysis in Fig 3. (b) Functional activations based on comparisons within the scream types from post-experimental classifications. (c) Functional activations for positive screams compared with neutral and negative screams. Threshold p = 0.005 voxel level, cluster size of k = 42 (corrected p = 0.05 at cluster level). (TIF) [file pbio.3000751.s007.tif]

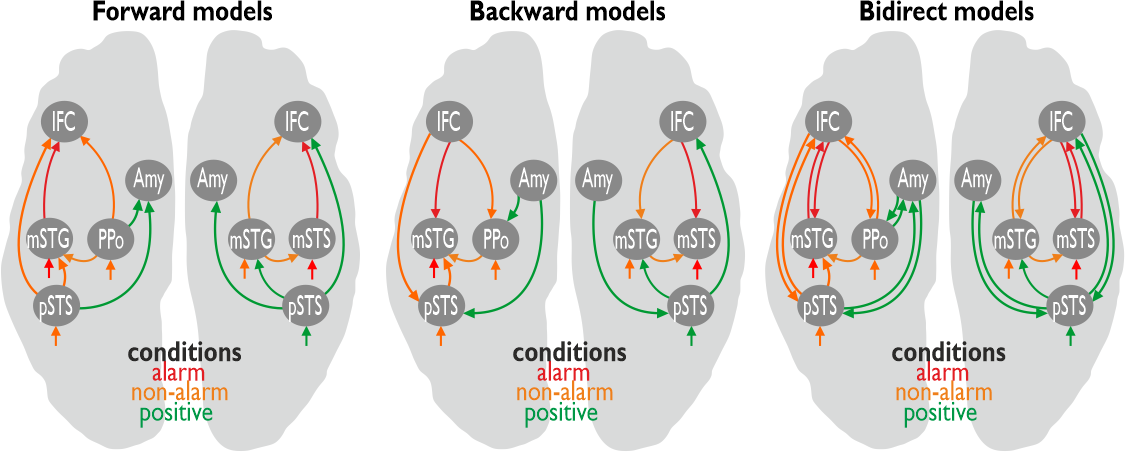

Supplement: S4 Fig — Three model families were created for both the left and the right hemisphere, including a family of forward models (left), a family of backward models (middle), and a family of bidirectional models (right). While all families were identical on the local connection of subregions in the auditory cortex, they differed in terms of connections from auditory subregions to the amygdala and the IFC. Forward models included connections from the auditory cortex to the IFC and the amygdala, backward models included connections from the IFC and the amygdala to the auditory cortex, and bidirectional models included bidirectional connections between the auditory regions and the amygdala and IFC. These connections defined the A matrix for DCM and were constant across the permutation across the model space derived from the B matrix. The B matrix defined the modulation connection by experimental conditions, which are color coded in the figure. The alarm condition (red) included only trials with alarm screams (pain, fear, anger), the non-alarm condition (orange) included only trials with non-alarm screams (pleasure, sadness, joy), and the positive condition (green) included only positive screams (pleasure, joy). The input C matrix (small arrows) defined the condition that provides input into the connectivity matrix, and we defined the input to each of the auditory subregions on the basis of predominant activity (or deactivations) resulting from the group-level contrasts. (TIF) [file pbio.3000751.s008.tif]
