# Supplementary material for: Neurocognitive processing efficiency for discriminating human non-alarm rather than alarm scream calls
Source: PLoS Biol. 2021 Apr 13;19(4):e3000751. doi: 10.1371/journal.pbio.3000751 (PMC8043411; doi:10.1371/journal.pbio.3000751)
Supplement: S1 Table — The table lists all significant peak activations for contrasting scream categories against each other (a–n). All activations are threshold at p < 0.05 corrected at the cluster level. (PDF) [file pbio.3000751.s009.pdf]

S1 Table. Peak activations for group-level contrasts between scream categories.
The table (a-n) lists all significant peak activations for contrasting scream categories against each other. All activations are threshold at p<0.05 corrected at the cluster level.

			MNI coordinates		
Region	Cluster size	z value	x	y	z
**(a) scream > neutral**					
L middle temporal gyrus	44	4.06	-43	-7	-22
L caudate nucleus	72	3.81	-10	17	17
R pulvinar	73	3.11	17	-34	12
**(b) neutral > scream**					
R middle frontal gyrus	847	4.69	46	38	17
R precentral gyrus		4.15	47	9	29
R inferior frontal gyrus		3.37	42	21	27
L globus pallidus	66	3.77	-16	-3	5
L middle temporal gyrus	50	3.54	-46	9	-16
L precentral gyrus	43	3.07	-39	5	34
L inferior frontal gyrus		2.63	-33	9	32
**(c) alarm > neutral**					

**(d) neutral > alarm**					
R inferior frontal gyrus	1372	5.19	46	9	29
R middle frontal gyrus		5.13	46	31	22
R superior temporal gyrus	505	4.60	56	-20	-5
R superior temporal sulcus		3.92	47	-20	-5
L superior temporal gyrus	260	4.06	-61	-25	5
L superior temporal sulcus		3.65	-53	-27	-4
L precentral gyrus	319	4.02	-39	7	34
L inferior frontal gyrus		3.54	-48	10	35
L middle temporal gyrus	171	4.01	-46	9	-16
L planum polare		3.00	-43	19	-16
L middle frontal gyrus	95	3.58	-34	53	-2
R inferior frontal gyrus	48	3.47	30	48	-10
R orbital gyrus		2.88	25	41	-9
L globus pallidus	85	3.40	-16	-3	5
R striate area	109	3.29	10	-76	1
R middle frontal gyrus	53	3.18	29	56	3
**(e) non-alarm > neutral**					
L caudate nucleus	116	4.36	-10	17	17
L planum polare	58	3.98	-43	-7	-21
R pulvinar	283	3.90	18	-34	12
R caudate nucleus		3.72	27	-35	12
R inferior frontal gyrus	47	3.79	35	2	24
R superior temporal gyrus	58	3.61	63	-13	0

L parahippocampal gyrus	77	3.39	0	-51	-4
R ventral anterior thalamus	75	3.38	0	-13	13
L fusiform gyrus	61	3.35	-21	-58	-22
L medial dorsal thalamus	57	3.13	-5	-27	8
R substantia nigra	43	3.13	10	-24	-10
**(f) neutral > non-alarm**					
R middle frontal gyrus	155	3.80	46	38	17
L globus pallidus	43	3.66	-16	-3	5
L precentral gyrus	49	3.32	-38	-7	20
**(g) alarm > non-alarm**					

**(h) non-alarm > alarm**					
R superior temporal sulcus	2673	7.66	54	-24	-2
R superior temporal gyrus		5.05	68	-32	8
R inferior frontal gyrus	1437	5.05	42	9	27
R middle frontal gyrus		3.99	52	33	13
L superior temporal gyrus	1590	5.01	-61	-27	8
L inferior frontal gyrus		4.38	-44	21	-16
R occipital gyrus	2992	4.86	7	-75	-24
R fusiform gyrus		3.93	30	-51	-29
L inferior frontal gyrus	1552	4.57	-44	10	29
L precentral gyrus		3.77	-39	7	34
R middle temporal gyrus	174	4.42	44	-39	-16
R inferior temporal gyrus		3.71	49	-49	-19
L amygdala	173	3.16	-9	-8	-10
L hippocampal gyrus		3.11	-16	-13	-12
L substantia nigra		2.89	0	-18	-12
L hippocampal gyrus	54	3.74	-31	-12	-16
L pulvinar	93	3.72	-21	-30	7
L caudate nucleus		2.63	-24	-35	13
L subgenual gyrus	116	3.71	-7	21	5
R middle frontal gyrus	247	3.71	27	53	5
R middle frontal gyrus	54	3.47	39	46	18
R putamen	112	3.41	17	-7	8
R pulvinar	232	3.35	20	-30	10
R caudate nucleus		3.20	29	-37	10
L superior frontal gyrus	42	2.98	-5	41	39
**(i) positive > neutral**					
L caudate nucleus	95	4.14	-12	17	17
L medial dorsal thalamus	92	3.52	0	-13	13
R pulvinar	150	3.38	18	-34	12
R caudate nucleus		3.32	25	-37	12
R superior temporal sulcus	99	3.34	47	-37	5
R substantia nigra	85	3.32	10	-24	-10
R inferior colliculus		3.20	7	-32	-9

R superior temporal sulcus	48	3.15	37	-29	-4
			0	0	0
**(j) neutral > positive**			0	0	0
L precentral gyrus	130	4.23	-38	-7	20
R middle frontal gyrus	127	3.75	46	38	17
L middle temporal gyrus	100	3.67	-39	-59	-2
**(k) negative > neutral**					

**(l) neutral > negative**					
R middle frontal gyrus	1149	4.92	46	31	22
R inferior frontal gyrus		4.70	46	9	29
R superior temporal sulcus	390	4.02	56	-20	-5
R superior temporal gyrus		3.99	54	-7	-10
L middle temporal gyrus	155	3.97	-46	9	-16
L planum polare		2.86	-39	19	-14
L superior temporal sulcus	128	3.80	-51	-27	-2
L superior temporal gyrus		3.48	-61	-25	5
L cerebellum	50	3.77	-10	-75	-34
L globus pallidus	90	3.70	-16	-3	5
L precentral gyrus	100	3.44	-39	5	34
L inferior frontal gyrus		2.75	-48	10	35
**(m) positive > negative**					
R superior temporal sulcus	2763	7.08	54	-24	-4
R middle temporal gyrus		6.11	54	7	-12
R superior temporal gyrus		4.12	64	-44	13
R inferior frontal gyrus	964	4.61	54	33	10
R precentral gyrus		4.21	42	9	27
L superior temporal gyrus	1066	4.43	-63	-35	8
L superior temporal sulcus		4.22	-53	-7	-10
L amygdala		3.97	-29	2	-17
L planum polare		3.60	-41	4	-16
R middle temporal gyrus	59	4.35	44	-39	-16
R inferior temporal gyrus		2.97	49	-52	-19
L planum polare	148	4.30	-43	21	-17
L inferior frontal gyrus	461	4.07	-50	34	13
R cerebellum	653	4.04	17	-80	-33
L cerebellum	149	3.60	-9	-78	-36
R hippocampal gyrus	82	3.45	27	-29	1
R amygdala	55	2.91	30	-3	-17
L inferior frontal gyrus	172	3.27	-44	10	24
R cerebellum	64	3.24	32	-51	-27
R inferior colliculus	198	3.18	5	-32	-10
**(n) negative > positive**					
L precentral gyrus	182	3.38	-38	-7	20
L inferior temporal gyrus	223	3.33	-39	-59	0

L lingual gyrus	2.84	-24	-47	-2
