# Supplementary material for: Neurocognitive processing efficiency for discriminating human non-alarm rather than alarm scream calls
Source: PLoS Biol. 2021 Apr 13;19(4):e3000751. doi: 10.1371/journal.pbio.3000751 (PMC8043411; doi:10.1371/journal.pbio.3000751)
Supplement: S2 Table — (a) No functional activations were found for a positive parametric analysis, taking the rated alarm level of each scream into account. (b) Functional activations for a negative parametric analysis with the alarm level of each scream. All activations are threshold at p < 0.05 corrected at the cluster level. (PDF) [file pbio.3000751.s010.pdf]

S2 Table. Group-level peak activations for the parametric analysis including alarm ratings.
(a) No functional activations were found for a positive parametric analysis, taking the rated alarm level of each scream into account.
(b) Functional activations for a negative parametric analysis with the alarm level of each scream. All activations are threshold at $p<0.05$ corrected at the cluster level.

			MNI coordinates		
Region	Cluster size	z value	x	y	z
**(a) alarm (positive)**					

**(b) alarm (negative)**					
R superior temporal sulcus	2436	5.01	52	14	-17
R superior temporal gyrus		4.67	64	-10	-2
R planum temporale		4.20	58	-17	18
L parahippocampal gyrus	459	4.85	-21	-46	-7
L precuneus		3.69	-5	-54	5
L superior temporal gyrus	2297	4.57	-60	-8	-5
L superior temporal sulcus		4.24	-46	-41	8
R inferior frontal gyrus	396	4.38	29	58	-2
R middle frontal gyrus	577	4.37	34	36	37
R inferior frontal gyrus		4.11	42	27	27
R cingulate gyrus	694	4.26	13	27	27
L medial frontal gyrus		3.72	-4	43	37
R cingulate gyrus		3.55	10	21	39
L medial frontal gyrus	115	4.23	-10	34	22
R cerebellum	558	4.23	3	-73	-26
L inferior frontal gyrus	658	4.20	-53	12	32
L precentral gyrus		3.95	-36	5	34
L amygdala	57	4.18	-17	-17	-10
L middle frontal gyrus	287	4.17	-31	51	25
R fusiform gyrus	887	4.11	32	-54	-22
R parahippocampal gyrus		3.81	25	-34	-22
R occipital gyrus		3.56	29	-80	-12
L striate area	394	4.01	-9	-93	-2
L inferior temporal gyrus	421	3.97	-44	-56	-21
L fusiform gyrus		3.41	-36	-69	-14
L hippocampal gyrus	142	3.77	-36	-13	-22
L striate area	189	3.73	-7	-83	-12
R precuneus	286	3.70	12	-54	10
R inferior frontal gyrus	235	3.64	42	21	-7
L putamen	52	3.64	-24	-8	5
R middle frontal gyrus	88	3.60	35	46	25
R cerebellum	66	3.57	15	-56	-22
R putamen	179	3.57	20	-15	8
R globus pallidus		3.37	18	-8	3
R ventral lateral thalamus		3.13	12	-20	10
R inferior temporal gyrus	47	3.56	47	-69	-2
L putamen	45	3.53	-26	-12	15

R middle temporal gyrus	45	3.50	41	-59	3
R superior temporal sulcus		3.17	46	-56	5
R amygdala	81	3.46	29	-13	-12
R middle temporal gyrus	71	3.41	44	-37	-16
R inferior temporal gyrus		3.33	41	-30	-17
R middle temporal gyrus	102	3.40	51	-49	8
R parahippocampal gyrus	112	3.40	13	-42	-7
R hippocampal gyrus		2.93	18	-32	-4
L fusiform gyrus	48	3.28	-27	-47	-17
L insula	64	3.18	-33	-7	8
L middle frontal gyrus	73	3.11	-26	34	37
