# Supplementary material for: Neurocognitive processing efficiency for discriminating human non-alarm rather than alarm scream calls
Source: PLoS Biol. 2021 Apr 13;19(4):e3000751. doi: 10.1371/journal.pbio.3000751 (PMC8043411; doi:10.1371/journal.pbio.3000751)
Supplement: S3 Table — (a) Group parameter estimates (FDR corrected p-values in parentheses) resulting from Bayesian model averaging (BMA) of the estimated parameters for the C matrix (input matrix), the A matrix (intrinsic connectivity matrix), and the B matrix (connection modulation matrix) for the left hemispheric models of the bidirectional family (winning family). The left hemisphere dynamic causal models included 5 regions; for the A and B matrix, the table is organized such that columns are the origin of the connection and rows are the target of the connection. Cell entries denoted as “na” were connections in the A matrix, but since for the left hemisphere, a single model was the overall winner, BMA was applied only to this model that had 2 B matrix entries that were not part of the estimation process. (b) Group parameters for the right hemisphere. (PDF) [file pbio.3000751.s011.pdf]

S3 Table. Estimated connectivity parameters resulting from the DCM analysis.

(A) Group parameter estimates (FDR corrected p-values in parentheses) resulting from Bayesian model averaging (BMA) of the estimated parameters for the C matrix (input matrix), the A matrix (intrinsic connectivity matrix), and the B matrix (connection modulation matrix) for the left hemispheric models of the bidirect family (winning family). The left hemisphere DCMs included 5 regions; for the A and B matrix, the table is organized such that columns are the origin of the connection and lines are the target of the connection. Cell entries denoted as “na” were connections in the A matrix, but since for the left hemisphere, a single model was the overall winner, BMA was applied only to this model that had two B matrix entries that were not part of the estimation process. (B) Group parameters for the right hemisphere.

**(a) Left hemisphere**					
	**pSTS**	**mSTG**	**PPo**	**Amygdala**	**IFC**
**C matrix (input)**	0.1140 (<0.001)	0.0081 (0.6518)	0.0121 (0.6518)		
**A matrix (intrinsic connectivity)**					
pSTS				0.0140 (0.3658)	0.0797 (0.0269)
mSTG	0.0177 (0.3303)		0.0128 (0.6456)		0.0363 (0.0456)
PPo				0.0094 (0.6735)	0.0626 (0.0269)
Amygdala	0.0576 (0.0269)		0.0231 (0.3658)		
IFC	0.0222 (0.0456)	0.0048 (0.8599)	0.0415 (0.0545)		
**B matrix (modulation by condition**)	**Alarm screams**				
mSTG					na
IFC		0.2559 (0.6750)			
	**Non-alarm screams**				
pSTS					-0.1170 (0.8683)
mSTG	0.7236 (0.0086)		na		
PPo					0.0765 (0.7443)
IFC	0.1339 (0.7443)		-0.0088 (0.9485)		
	**Positive screams**				
pSTS				0.0147 (0.9485)	
PPo				0.2073 (0.6750)	
Amygdala	0.1881 (0.6750)		0.0397 (0.9485)		
**(b) Right hemisphere**					

	pSTS	mSTG	mSTS	Amygdala	IFC
**C matrix (input)**	0.0800 (0.0346)	0.0938 (0.0031)	0.0046 (0.8172)		
**A matrix (intrinsic connectivity)**					
pSTS				0.0139 (0.2166)	0.0387 (0.2166)
pSTS	0.0261 (0.1490)				0.0693 (0.0198)
mSTS		0.0340 (0.0327)			0.0338 (0.0458)
Amygdala	0.0571 (0.0198)				
IFC	0.0097 (0.6645)	0.0394 (0.1869)	0.0180 (0.1869)		
**B matrix (modulation by condition)**	**Alarm screams**				
mSTS					0.0576 (0.6412)
IFC			0.2353 (0.1760)		
	**Non-alarm screams**				
mSTG					0.1893 (0.1618)
mSTS		0.5756 (0.0001)			
IFC		0.0162 (0.8916)			
	**Positive screams**				
pSTS				0.0588 (0.1315)	0.0459 (0.6835)
Amygdala	0.3392 (0.0156)				
IFC	0.4292 (0.0070)				
