# Supplementary material for: Neurocognitive processing efficiency for discriminating human non-alarm rather than alarm scream calls
Source: PLoS Biol. 2021 Apr 13;19(4):e3000751. doi: 10.1371/journal.pbio.3000751 (PMC8043411; doi:10.1371/journal.pbio.3000751)
Supplement: S1 Text — (PDF) [file pbio.3000751.s012.pdf]

Statistical analysis for data presented in S1 Fig.

Fig. S1a
Accuracy rate (upper plot): Scream types (7 levels): $F_{6,150}$=9.419, $p<0.001$, η^2=0.22; three major categories (neutral, non-alarm, alarm); $F_{2,50}$=17.799, $p<0.001$, η^2=0.26
False alarm rate (mid lower plot): Scream types (7 levels): $F_{6,150}$=19.406, $p<0.001$, η^2=0.41; three major categories (neutral, non-alarm, alarm); $F_{2,50}$=79.305, $p<0.001$, η^2=0.69
Arousal rating: Scream types (7 levels): $F_{6,150}$=70.842, $p<0.001$, η^2=0.42; three major categories (neutral, non-alarm, alarm); $F_{2,50}$=105.780, $p<0.001$, η^2=0.51

Fig. S1b
Accuracy rate: Scream types (7 levels): $F_{6,150}$=1.895, $p=0.117$, η^2=0.06; three major categories (neutral, non-alarm, alarm); $F_{2,50}$=2.231, $p=0.121$, η^2=0.05
False alarm rate: Scream types (7 levels): $F_{6,150}$=23.303, $p<0.001$, η^2=0.46; three major categories (neutral, non-alarm, alarm); $F_{2,50}$=60.942, $p<0.001$, η^2=0.66
Arousal rating: Scream types (7 levels): $F_{6,150}$=51.065, $p<0.001$, η^2=0.38; three major categories (neutral, non-alarm, alarm); $F_{2,50}$=84.558, $p<0.001$, η^2=0.48

Fig. S1c
Accuracy rate: Scream types (7 levels): $F_{6,150}$=9.160, $p<0.001$, η^2=0.13; three major categories (neutral, non-alarm, alarm); $F_{2,50}$=16.596, $p<0.001$, η^2=0.13
False alarm rate: Scream types (7 levels): $F_{6,150}$=29.761, $p<0.001$, η^2=0.43; three major categories (neutral, non-alarm, alarm); $F_{2,50}$=57.719, $p<0.001$, η^2=0.53
Arousal rating: Scream types (7 levels): $F_{6,150}$=51.918, $p<0.001$, η^2=0.45; three major categories (neutral, non-alarm, alarm); $F_{2,50}$=37.511, $p<0.001$, η^2=0.23
