# Supplementary material for: Neurocognitive processing efficiency for discriminating human non-alarm rather than alarm scream calls
Source: PLoS Biol. 2021 Apr 13;19(4):e3000751. doi: 10.1371/journal.pbio.3000751 (PMC8043411; doi:10.1371/journal.pbio.3000751)
Supplement: Attachment — Submitted filename: SFruhholz_reponse_to_reviewers.pdf [file pbio.3000751.s013.pdf]

Reponses to comments by the reviewers
PBIOLOGY-D-20-00286R1

REVIEWERS' COMMENTS:

Reviewer #1: This manuscript presents data from four sufficiently powered experiments investigating human scream calls using perceptual ratings, classification of acoustic parameters and functional magnetic resonance imaging. Fruhholz and colleagues studied whether human screams necessarily reflect alarm signals and found that listeners judged screams associated with pleasure, sadness or joy less alarming compared to screams who have been recorded while speakers intended to convey anger, fear or pain. Classification of alarm screams as belonging to a specific category took listeners longer and was less correct compared to non-alarm or neutral screams, although classification of acoustic parameters using SVMs was similarly efficient for non-alarm and alarm screams. fMRI during a gender identification task using the same stimuli revealed diminished activity for alarm compared to non-alarm screams in frontal and temporal cortex. The authors' interpretation of findings in the sense of a "superior efficiency" for non-alarming screams is not fully supported by evidence. The findings could also indicate better discrimination of scream subtypes for non-alarm than alarm screams only in the context of a discrimination task, which could be ecologically more valid for non-alarm vs. alarm screams.
[Response] We thank the reviewer for the constructive and valuable comments on our manuscript. We tried to thoroughly revise our manuscript along the major comments.

A particular strength of the manuscript is the use of a broad range of screams with different affective valence and the use of a well-defined stimulus set in a variety of experiments. The main limitation of this manuscript, however, is the choice of tasks in the various experiments and the way tasks influence findings is discussed.
[Response] we agree that the choice of task is a relevant feature of our manuscript. For the revised manuscript we tried to better introduce this task issue in many party of the manuscript (e.g. the abstract). See the major specific comments and our responses below.

While participants identified and classified screams as either neutral, pleasure, sadness, joy, pain, fear or anger screams in the behavioral experiments, the fMRI data are based on data during gender identification. RTs are extremely long (1.5 -2.5 s in the 7AFC and .9 to 1.3 in the 2AFC) and I believe it is not reported whether participants were instructed to respond as quickly as they can.
[Response] Yes, participants were instructed to responds as quickly as possible without sacrificing accuracy. This missing information is now added to the manuscript, p23: “Participants were asked to respond as quickly as possible, but still trying to make an accurate decision”.

Please also note that for the 7AFC we used a mouse-based response task that seems responsible for slightly longer response latencies, p23: “Decision options were presented on the screen in a circular arrangement, each at ~5° visual angle from the center of the screen. Participants had to respond by moving the mouse cursor from the middle of the circle (i.e. mouse cursor position was reset to the middle of the circle after each trial) to one of the response options and pressing the left mouse button with the right index finger”. For the 2AFC, we have the impression that this is in the normal range of response latencies for such tasks.

However, the higher false alarm rate for alarm compared to non-alarm screams suggests indeed impaired classification of alarm compared to non-alarm screams by listeners. The problem is that the authors interpret response speed when classifying screams as either neutral, pleasure,

sadness, joy, pain, fear or anger screams as "neurocognitive processing efficiency". The literature is full of examples with faster reaction times for alarm signals compared to non-alarming signals. Reaction times depend highly on the task. The current finding of longer reaction times during classification of alarm screams is interpreted as reduced processing efficiency compared to non-alarm or neutral screams. I tend to disagree with this interpretation. The alarming nature of screams may interfere with the classification task, asking for suppression of these automatic processes, which may potentially explain reduced neural activity, negative modulation of temporo-frontal effective connectivity and longer reaction times. This interpretation could be supported by reporting reaction times for ratings of the alarming quality of stimuli in Exp. 1. Alternatively, discrimination may be more ecologically valid in the evaluation of non-alarm compared to alarm screams.

[Response] In cognitive psychology and in experimental psychology as well as in the computational modeling of RTs and RT-distributions for decision tasks (e.g. Ratcliff's drift diffusion model and follow-up modelling approaches), RTs for many years been thought as a proxy to neurocognitive processing efficiency. In line with this tradition, we also think that RT in our studies is a proxy to neurocognitive processing efficiency (otherwise this would invalidate many years of experimental psychological studies). We however agree that there is a long line of studies (e.g. the emotional Stroop task), where processing the task-irrelevant affective quality of a stimulus distracts from the relevant task. But in our study, the affect quality was the task-relevant and not task-irrelevant dimension.

Although we feel that the reviewer's suggestion is somehow interesting ("… The alarming nature of screams may interfere with the classification task .."), it already contradicts itself within this comment. The comment says that "The literature is full of examples with faster reaction times for alarm signals compared to non-alarming signals", so obviously in previous studies the alarm quality did NOT distract from the classification task (which would have resulted in longer RTs for alarm signals). So if the previous literature is correct (i.e. faster response to alarm signals), the suggestion by the reviewer seems wrong (i.e. there is no distraction in this sense).

Furthermore, if this distraction would lead to a "… suppression of these automatic processes, which may potentially explain reduced neural activity, negative modulation of temporo-frontal effective connectivity and longer reaction times". Neural suppression is a costly process, one brain system has to suppress another one (or suppress itself); so suppression should lead to higher and not to lower activity.

Finally, concerning the notion that "… discrimination may be more ecologically valid in the evaluation of non-alarm compared to alarm screams …", we think it is largely the opposite that seems true. Discrimination between non-alarm is important, but has less relevance if sometimes misclassified (e.g. confusion of joy and pleasure has no dramatic consequences for the listener). However, discrimination within alarm screams can be lifesaving (e.g. mistaken an aggressive anger scream for a pain scream might cause you big harm, because you then tend to approach rather than to avoid the angry person).

Reaction times for alarm ratings did not show differences between neutral, non-alarm, and alarm screams ($F_{2,44}$=3.015, p=0.084); see below.

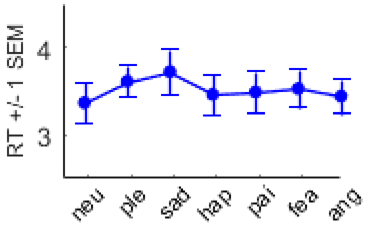

I recommend that the abstract and the discussion more clearly state the task-dependency of findings and reduce generalization beyond the studied tasks to a minimum. In the same line, "Neural priority for non-alarm-screams" or "behavioral advantage for non-alarm screams" are misleading overgeneralizations that should be avoided.

[Response] We agree with the reviewer that we should more highlight the task-dependency of the data. Most importantly, the variation of the task with an important feature of the behavioral experiments (experiment 2 and 3); for these experiment we kept the stimulus material constant, but varied the task based on certain decision that people also do in daily environments. We also highlight the fact that there was a difference in task between the behavioral experiments and the fMRI experiment (experiment 4).

In the abstract we now mention the task-dependency and tried to avoid any overgeneralizations. Concerning these overgeneralizations, we still see that our data show that alarm screams show overall less behavioral processing efficiency, and the neural effects show that the human brain is more tuned to process non-alarm screams. Given these differences, we think we should mention them in the abstract, and give some boarder perceptive on the relevance of our data (which is what every experimental study usually does). We now avoid the terms “neural priority” and “behavioral advantage”.

P16: “One further specific note concerns the various combinations of two specific scream types in experiment 3. Some of these combinations might only rarely occur in daily life, but for completeness we include any possible combination here. For example, specific combinations of non-alarm and alarm screams might occur less frequently in daily life (e.g. joy and anger screams), but there nonetheless certain contexts that include co-occurrences of non-alarm and alarm screams (e.g. a happy social gathering, where an interactions between two people suddenly turns violent because of an incident)”.

P15: “We have to note that we used the exact same stimulus material in experiment 2 and 3, but critically changed the decisional tasks that participants had to perform. In experiment 3 we asked participants to only decide between two possible scream types, and found the highest reaction times and error rates while discriminating between alarm scream”.

P17: “We have to note that all neural activations and specifically all connectivity results were obtained while participants performed a non-emotional gender decision task, and thus participants were not explicitly focusing on the affective quality of the screams. However, the implicit processing of the affective quality of voice signals has been shown by many previous studies [1,2], and given that the gender task was largely unrelated to the affective and alarming quality of the screams types, we think that the neural activations and connected patterns largely reflect the neural dynamics for generic scream processing and scream discrimination”.

P18: “A different picture of scream calls seems to emerge when investigated in humans, such that human listeners overall respond more quickly, more accurately, and with higher neural sensitivity to non-alarm and positive scream calls, which seem to have a higher relevance in human sociobiological interactions [3–5]. There seem some exceptions from this overall pattern of scream recognition in humans, but across many psychoacoustic, behavioral, perceptual, and neural effects quantified here, alarm screams often show less neurocognitive processing efficiency than non-alarm screams. Alarm cream categories only have some primacy during misclassification of other scream types, which might be a safety choice under conditions of decisional uncertainty. And this safety choice might be shared with other non-human species that use screams in their vocal repertoire”.

An important point that the authors missed in their discussion is that non-alarm and alarm calls usually do not co-occur. Rather, they occur in very different contexts and it may therefore be less important to differentiate them based on their acoustics only. The discrimination task between alarm and non-alarm screams in Exp. 3 may be less ecologically valid than judging the alarming

quality and the spatial location of the screaming person. Adding the same analyses for RT and sensitivity for the rating of alarm quality in Exp. 1 may help (see above).
[Response] Although non-alarm screams might not as frequently co-occur than non-alarm and alarm screams, there are however certain occasions where this could happen. A less frequent co-occurrence of non-alarm and alarm screams that does not imply that this is less ecologically valid. We included notion about this issue in the discussion section, p15: "One further specific note concerns the various combinations of two specific scream types in experiment 3. Some of these combinations might only rarely occur in daily life, but for completeness we include any possible combination here. For example, specific combinations of non-alarm and alarm screams might occur less frequently in daily life (e.g. joy and anger screams), but there nonetheless certain contexts that include co-occurrences of non-alarm and alarm screams (e.g. a happy social gathering, where an interactions between two people suddenly turns violent because of an incident)".

The authors do not comment on the different tasks in the behavioral and fMRI experiments. There are considerable differences in the mechanisms underlying gender identification and scream classification. I understand that the authors wanted to use a task that is orthogonal to the parameters of interest, but I am not sure that it actually is orthogonal (again, could there be an interaction between the alarming quality of the signal and the task?).
[Response] This gender task as included in the fMRI study here was used in many previous studies [1,2] based on the reasoning that it is orthogonal to the affect dimension of stimuli. We included the behavioral data of the gender task now in the manuscript, see Fig. S2. The statistical analysis were included on p11: "Reactions times did not differ between all 7 scream types ($F_{6,174}$=2.371, p=0.094) nor between the 3 major categories of neutral, non-alarm, and alarm screams ($F_{2,58}$=0.756, p=0.428). Error rate was different between all 7 scream types ($F_{6,174}$=7.280, p<0.001, η^2=0.10), but not between the 3 major categories ($F_{2,58}$=2.177, p=0.132)".

As the data show the RTs were uninfluenced by the scream types. We found that error rates difference between all 7 screams types, but nit between the major categories of neutral, non-alarm, and alarm screams. Thus, there was no interaction between the alarming quality of the signal and the task as supposed by the reviewer.

The second important question concerns the consequences of the standardization procedure. Could it be that the standardization procedure affected alarm screams to a larger degree compared to the other screams? Please provide the details on the acoustic and perceptual consequences.
[Response] The background for the standardization procedure is that in precise psychoacoustic and neuroimaging experiment, one has to carefully match stimuli for features that might induce significant differences themselves. We know that stimulus duration and stimulus intensity can significantly drive brain activity (especially in auditory cortex), and in order to avoid such confounding effects we opted for the standardization (also for the behavioral experiments) being aware that some stimuli might have sounded less "natural". But we think the precision in the experimental setup outweighs the effects introduced by standardization. Especially intensity normalization was introduced, because recording intensity can be quite different between speakers, which largely depends on the microphone-to-mouth distance. Furthermore, this standardization procedure was used in many previous neuroimaging studies on emotional voice perception to avoid confounding effects.

84 screams were selected from 420 screams. How robust were findings against this selection procedure?
[Response] The 84 screams were selected based on statistical criteria. This is documented on p20: "From the acoustic scream recordings of experiment 1, we selected 84 screams (3 male, 3

female speakers) with 2 instances of screams per category. Stimuli were selected from the results of the perceptual assessment of screams in experiment 1, such that no significant differences in the recognition rate across scream types ($F_{1,6}$=1.895, p=0.085) were found for this selection. Mean arousal level differed across all 7 scream types ($F_{1,6}$=51.065, p<0.001), across the 6 generic screams ($F_{1,5}$=11.647, p<0.001), and between neutral, alarm, and non-alarm screams ($F_{1,2}$=77.645, p<0.001)". Thus, the most important criteria for the selection was that each category of screams for the selected stimuli had an equal level of base recognition rate. This is necessary, because any performance different in the 7AFC and the 2AFC should not be confounded by differences in the base recognition rate; otherwise the behavioral would not be interpretable. We could of course have selected other 84 screams, but at the risk the base recognition rate would not have been equal, and thus we would have introduce a confounding feature.

Could it be that the inclusion of the arousal level as a covariate of no interest in the fMRI analysis explained away variance otherwise explained by the alarm screams? Figure 4 suggests that this was the case. Please provide the correlation between regressors. I understand the reason why to include this covariate, but it would be good to know in which way this choice influenced the fMRI results.
[Response] For the analysis shown in Fig. 4, we included both arousal (regressor of no interest) and alarm ratings (regressor of interest) as covariates in the analysis, both regressors were not orthogonalized. Both regressors correlated differently for each participant (based on individual arousal rating) in a range r = -0.0337 – 0.6713 (p = 0.8403 – 0.0001). Given that there was no uniform correlation across participants, and given that activity in Fig. 4 is derived from alarm ratings with arousal as regressor of no interest, we infer that activity shown in Fig. 4 is largely based on alarm ratings independent of arousal ratings. Thus, arousal as covariate in the original contrasts is unlikely to have influenced the activations.

The DCMs do not seem to test a specific hypothesis, which is a shortcoming, because a specific hypothesis could have indeed been formulated. Instead, the authors chose to test thousands of different models and identify a winning model based on fMRI data during gender identification.
However, some important information, particularly on model specification, is lacking. Is the lack of a driving input into the amygdala data-driven or pre-specified?
The data in the left and right hemisphere seem to stem from different groups (were the two excluded participants identical for the left and right hemisphere)? In case they were not identical, I suggest removing both subjects from both analyses.
In the current version of the manuscript, the DCMs do not add much to the overall story. Maybe emphasizing what the found modulations by scream type indicate in the context of gender identification can help further improve the manuscript. Again, I believe that the task context is critical and should not be neglected. Gender identification may be a more important issue during social, non-alarming screams while alarm screams have different primary consequences.
[Response] DCM modelling is commonly done by including informed restriction to the broad model space that can be tested. These limitations are introduced based on hypothesis on parameters that are supposed to influence the neural network analysis. These limitations can be very strict in the sense that one tests and fits only a single model to data and test which input, connections, and modulations become significant. Given that this is rarely the case and seems also very uninformative about the data, researchers choose to either test a set of models or testing the whole possible model space within the fixed restrictions. Both approaches are found in the literature and are valid approaches given general DCM guidelines [6]. While the formed approach has the computational advantage of testing only a limited set of models, it always has the shortcoming of missing other models that might have fitted data significantly better. This is why some researcher take the second approach and define the winning model best on the best fit.

This approach is unbiased to a certain model section that might be biased be the researchers believe about what to find.

We introduced the following limitations that defined the model space that was tested in our study: (1) we fixed the input C matrix for all models based on the functional activity and specificity of a certain AC region for specific scream types; (2) we fixed the A matrix connections based on known functional and structural connections between the brain regions; (3) we fixed the B matrix by defining which experimental was allowed to modulate a certain connection; all other parameters of the B matrix varied across models, and based on this variation we selected the best fitting models.

The lack of driving input to the amygdala is pre-specified given that auditory input is more likely to be processed by AC regions [7]. This is is now better described in the manuscript, p28: "For the left hemisphere, the input C matrix included non-alarm trials to the pSTS and the PPo, as well as alarm trials to the mSTG, while for the right hemisphere, the C matrix included positive trials to the pSTS, non-alarm trials to the mSTG, and alarm trials to the mSTS. This input C matrix was fixed for all models and only included subregions of the auditory cortex as potential regions for experimental input, but we estimated the strength and significance of these driving inputs for each model".

Concerning the exclusion of participants from the DCM analysis, this is quite common to DCM analyses, given that some subject often show outlier parameters. We here excluded n=2 subject per hemisphere, and these were not the same subjects. These subjects had unreliable values in certain ROIs probably based on the fact that the ROIs were too close to the brain/CSF boundary, which often leads to unreliable estimates. We of course thought about the option to exclude all four subjects from all DCM analyses, but were afraid that the resulting DCM results from n=20 would be too far away from the n=24 sample activations. With the current analysis, we have n=22 subjects for each hemisphere, which we think renders the DCM results closer to the original brain activations. We think that keeping the analysis sample closer to the entire sample is very crucial to keep the connection between the patterns of activations and the connectivity patterns.

Concerning the task effects and the additional information of the DCM results to the manuscript, we believe that the DCM data add important information to the manuscript. First, as outlined below, the gender task was not systematically related to the scream categories, and thus the DCM results should also be largely unaffected by the gender task, Second, we agree that mentioned the task is critical to the data. This gender task has been reliably used in many previous studies [1,2], and these studies refer to an implicit processing of the emotional quality of voices. This incidental processing has been shown in many studies, and is a valid way to investigate emotional processing in many fMRI studies.

To highlight this issue, we now introduce this notion in the manuscript, p12: "The task was introduced to maintain the attention of the participants to the experiment, and is often referred to as implicit but still strong processing of the affective quality of the stimuli that leads to consistent brain activations [1,2]". See also p17: "We have to note that all neural activations and specifically all connectivity results were obtained while participants performed a non-emotional gender decision task, and thus participants were not explicitly focusing on the affective quality of the screams. However, the implicit processing of the affective quality of voice signals has been shown by many previous studies [1,2], and given that the gender task was largely unrelated to the affective and alarming quality of the screams types, we think that the neural activations and connected patterns largely reflect the neural dynamics for generic scream processing and scream discrimination".

Are you sure that the text on page 7, second paragraph is correct? The Figure 2a indicates that neutral screams are classified as rapidly as joy screams compared to all other screams that are classified more slowly. Please add the post hocs for the first ANOVA (first paragraph page 7) and an illustration of the grouped data (second paragraph).

[Response] Yes, the text in general is correct, we just mixed up one word that was also mentioned be reviewer #2; this was corrected, p8: “with non-alarm (p=0.032) and alarm screams (p=0.026) having SLOWER mean reaction times than those for neutral screams, and non-alarm screams being classified faster than alarm screams (p=0.026)”. The posthoc test for the upper paragraph was added, and the grouped data for the lower paragraph was added as mean line (dashed line) in the figures, see Fig. 2a.

P7: “Comparisons across non-alarm and alarm screams showed that joy scream were classified faster than all alarm screams (all p’s<0.001) and pleasure being classified faster than pain screams (p=0.013). Only sad screams were classified slower than anger screams (p=0.010). Postdoc tests for the performance accuracy showed that neutral screams had better a classifications rate than the negative screams (all p’s<0.001), but not compared to the positive screams (all p’s>0.103); within the non-alarm screams, sad screams differed from pleasure (p=0.034) and joy screams (p<0.001), while alarm scream did not differ in performance accuracy (all p’s>0.142). Comparisons across non-alarm and alarm screams showed that joy screams were more accurately classified than anger (p=0.004) and pain screams (p<0.001)”.

Given these posthoc tests, there still seems the general pattern that alarm screams have longer classification times and more errors, but there are exceptions. We now include notions of these exceptions in the manuscript, p14: “There were some exceptions to this general observation that alarm scream were classified slower and with higher error rates. For example, sad screams were classified slower when specifically compared to anger screams; furthermore, the performance for joy screams had large effects on the mean performance during non-alarm scream classifications. But when quantifying the d prime measures as an indicator of perceptual sensitivity as well as the false alarm rate, the general observation of a lower processing efficiency of alarm compared to non-alarm scream was largely confirmed”.

The behavioral results of the gender identification task during fMRI seem to be missing. Was this task equally influenced by the alarming quality of the stimuli?

[Response] We included the behavioral data of the gender task now in the manuscript, see Fig. S2. The statistical analyses were included on p11: “Reactions times did not differ between all 7 scream types ($F_{6,174}$=2.371, p=0.094) nor between the 3 major categories of neutral, non-alarm, and alarm screams ($F_{2,58}$=0.756, p=0.428). Error rate was different between all 7 scream types ($F_{6,174}$=7.280, p<0.001, η^2=0.10), but not between the 3 major categories ($F_{2,58}$=2.177, p=0.132)”. There seemed no relationship to the alarm level of screams.

Acoustic analyses: There seems to be something specific about the joy screams, because the modulation spectrum largely lacks differences to neutral screams. Could this be a result of the standardization procedure? The authors could explore the SVM parameters to better understand the contribution of specific acoustic features to classification. Why was loudness included as a feature in case this parameter was standardized?

[Response] We agree that joy screams showed less difference to the neutral screams compared to the other screams, but still they showed a significant difference in the frequency ranges analyzed here based on p-value maps of the MPS resulting from a permutation test. This is also shown in Fig. 1b, lower row.

Data availability: Please deposit the anonymized dataset at an appropriate repository (see Instructions for authors).

[Response] We carefully checked the instructions, which mentions that a data availability statement should be included about how data can be made available. We included the statement that data can be shared based on reasonable request to the corresponding author. This seemed a valid and accepted option during the submission process. We have to note that at the time when the data were acquired, Swiss law and ethics regulation did not allow to deposit data in public

repositories without full and signed agreement by participants. Given that we do have this agreement only by a few participants, and intermediate solution in accordance with Swiss ethics guideline is to share date based on reasonable request.

Statistics: The f values in Exp. 1-3 are large, the error bars are surprisingly small. I wonder how exactly the data were entered into the analyses. How did you deal with normality, non-sphericity and interindividual variability? Please plot the individual datapoints.
[Response] The F values resulted from fitting a repeated measure model with an within-subject design to our experimental data; data were entered into the rmANOVAs as subject wise means per condition: the F values resulted from this analysis, and this is how they are (see below). The size of the error bars is a matter of scaling the y-axis. We for now avoided putting also single subject point into the graphs, because they are already dense in terms of display items, and would become largely unreadable. Non-sphericity was corrected using GG corrections in case the Mauchly's test was significant. Normality was assumed for all parametric test, and rmANOVAs are usually quite resistant against non-normality. Interindividual variability is expected (as in every experimental psychological study) and is already dealt with the within multi-level random effects models used here based on distribution statistics.

Example of resulting F values:

	SumSq	DF	MeanSq	F	pValue	pValueGG	pValueHF	pValueLB
(Intercept)	1.1249e+05	1	1.1249e+05	781.25	1.1081e-18	1.1081e-18	1.1081e-18	1.1081e-18
Error	3167.7	22	143.99					
(Intercept):type	33770	2	16885	227.63	6.2043e-24	1.915e-17	3.6624e-18	4.3696e-13
Error(type)	3263.7	44	74.176					

Reaction time plot with another scaling of the y-axis shows the error bars:

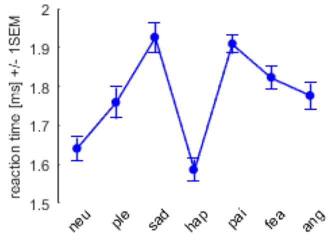

Minor points:

The references to panels of Figure 1 in the text are wrong.
[Response] This was changed, and the references to panels of Fig. 1 are now correct.

Please mention on page 9, first paragraph, how "negative screams" were defined.
[Response] "Negative screams" were defined on their negative affective valence, which is based on a common classification of emotions along the valence dimension. This is described in the last sentence of the introduction, and p10 we now re-introduce this distinction: "… (i.e. screams with a negative affective valence) …"

Please define the 4 levels for the Oneway ANOVA on page 9.
[Response] The 4 levels refer to the broader categories of combining scream types that are also shown in Fig. 1b: neutral and screams, within non-alarm, within alarm as well as alarm with non-alarm. This is now also clarified in the manuscript.

Page 10 suggests that fMRI was performed during scream call recognition, which was not the case. Please specify the task here.
[Response] This is now better described on p11: "To identify the neural dynamics of scream call processing in this network, we asked humans to listen to the same selected 84 screams as in experiments 2 and 3. During listening to these scream calls, participants performed an orthogonal gender decision task on the screams; the task was introduced to maintain the attention of the participants to the experiment".

The modulation power spectrum should be consistently abbreviated "MPS".
[Response] This was changed throughout the manuscript and in the figures.

Figure 3 shows neural activity, not neural mechanisms.
[Response] Was changed.

The coverage of the partial volume should be indicated in the Figures.
[Response] The partial volume space is now indicated in Fig. 1a

---

Reviewer #2: I found this manuscript very interesting and nice to read. It provides novel insights into the field of non-verbal vocal communication in humans. For instance, it shows that 6 types of screams can be identified based on their acoustic structure, and that these types can be associated with a variety of emotions, ranging from negative to positive and from low to high arousal. Interestingly, in terms of perception, listeners do not seem to be able to categorise between the three types of alarm screams (pain, fear and anger). Finally, there seems to be higher neural signal and connectivity in perceivers of non-alarm compared to alarms screams, which is a surprising result.
[Response] We thank the reviewer for this overall very positive evaluation of our manuscript and the useful comments, which really much helped to improve the manuscript.

However, regarding the claim that the authors are making about the fact that alarm screams are responded the slowest and associated with the lowest perceptual sensitivity, suggesting that alarm screams have inferior efficiency in cognitive processing compared to non-alarm calls, I would have an alternative, maybe more biologically relevant hypothesis to suggest, which could explain these surprising results (see below). In addition, I have some suggestions on how to improve the presentation of the results and a few other more specific comments, notably on the methods.
[Response] Thanks for the suggestion on how to improve the manuscript. Concerning the suggested alternative hypothesis, we give more detailed responses below.

General comment:

My main concern is your interpretation of the results obtained in experiments 1 and 2. I could suggest an alternative explanation, which would make more sense concerning the nature and function of screams. These two experiments suggest that people really have difficulties in differentiating between pain, anger and fear screams. As a result, your measurement of 'reaction time' might not accurately reflect how fast people reacted (e.g. got activated or attentive - behavioural reaction), but, if I understand correctly, the latency to classify/discriminate the

screams according to the 7 types. This measure thus in fact reflect how fast participants performed this seemingly difficult classification between the three categories of alarm screams.

You might have obtained a very different response if you had asked participants to choose simply between 'alarm' or 'non-alarm', although you might still get some misclassification of non-alarm screams as alarm, because the costs incurred by a failure in identifying an alarm context should be higher than the costs of misidentifying an non-alarm scream.

Your second experiment might not have solved this issue, as participants had to select a sound as 'pain' vs alternative, 'anger' vs alternative or 'fear' vs alternative (instead of 'alarm' vs 'non-alarm'). Participants might thus have hesitated about whether the sound they hear is really fear, pain or anger.

In any case, a misclassification of many screams as alarm screams, suggest that these sounds indeed have a high threat signalling nature. It could be that the function of the three types of alarm screams is to attract attention and trigger a fast stress response, independently of their actual type (indiscriminately), since in situation of danger, there might be no time for higher cognitive processing. This higher attention could then be followed by an evaluation of the situation and the integration of contextual cues, which would then allow receivers of the screams to discriminate between fear, pain and anger situations.

[Response] The reviewer raises an interesting hypothesis here that we quickly like to discuss here in relationship to our data. The data first show that the alarm screams take longer to classify, for each alarm scream separately (experiment 2). Furthermore, alarm screams include higher RTs to discriminate them from each other, and people make also more errors when they need to discriminate alarm screams (experiment 3). The arousal level (“stress response”) is almost the same for non-alarm and alarm screams, at least for sad, joy, pain, and fear (Fig. S1); so non-alarm screams can elicit the same kind of arousal than alarm screams, such that the high stress response seems not exclusive to alarm screams.

The reviewer proposes a “more biologically relevant hypothesis” (see comment above), but we think that we kindly need to disagree that this alternative hypothesis is biologically more relevant. We agree that alarm screams might elicit arousal to respond quickly to potential threats. But we disagree that this arousal state is “indiscriminate”; if this would be true, it would put the organism into a life threating situation. As we have noted in a response to a comment to reviwer#1, discrimination within alarm screams can be lifesaving (e.g. mistaken an aggressive anger scream for a pain scream might cause you big harm, because you then tend to approach rather than to avoid the angry person/source). So if alarm screams are arousing and elicit stress, they do so for a quick assessment (and classification) for what kind of threat is present. An alert state of an organisms (i.e. wide eyes, broad attention) is made to more accurately sample sensory information for a better classification of the context. And organisms needs to decide quickly if a stream results from pain (help the other person; you do not need to run away from a person screaming out of pain) or from anger (run away from the angry person).

In response to reviewer #1 we also quantified now the RTs to the alarm ratings presented in Fig. 1c. This is similar to the suggestion raised in the next comment by reviewer #2 about doing an experiment with only alarm/non-alarm judgements. If people would be faster for a simple alarm judgement, they should show faster RTs in the alarm rating. This was not the case.

Furthermore, we also have to note that in the discussion of our data, one should not only focus on RTs. In experiment 1 we analyzed data in four different levels: RTs, accuracy, false alarm rate, and d primes. The reviewers suggestion about a stress response might explain increased RTs for alarm screams, but it does not explain higher error rates for alarm screams; if people take longer to classify alarm screams (“higher cognitive processing”), it does not explain why they make more errors (if people take longer to process sensory information, the usually get better in classifying the stimulus). Increased RTs and higher errors (false alarms) combined lead to lower d prime values; lower d primes value indicate lower perceptual sensitivity. And this all happens with the background that on an acoustic level all scream types can be clearly distinguished (Fig. 1d).

Finally, if the initial physiological activation of the reviewer would be valid, we problem also would see higher brain activity and especially limbic/amygdala activity for alarm screams, which trigger the fast stress response. This was not the case, instead positive screams activated the brain and the amygdala more than alarm screams. Overall, we thus feel that the fast stress response hypothesis suggested by the reviewer is rather unlikely; and the data still indicate that alarm scrams are worse in terms of processing efficiency.

We included a notion about the suggested hypothesis now in the manuscript, since we feel it is very worth to discuss, p10: “There might be the possibility that alarm screams do not need to be discriminated very quickly, because the only need to activity the perceiving organisms to indiscriminately respond to any potential threat. While this could explain the increased classification times for alarm screams (i.e. respond first, and then discriminate), it does not explain the higher error rates, which still point to a classification and discrimination disadvantage for alarm screams”.

I think that your results clearly suggest that non-alarm calls have a higher discriminability advantage compared to alarm screams, because in a social context, it might be important for humans to tell joy, sadness and pleasure apart for example, while when hearing alarm calls, it might be more important to react quickly (e.g. run away), than to discriminate between anger, fear and pain. However, I would rephrase your interpretation of alarm screams having 'inferior processing efficiency' or 'behavioural disadvantage' compared to non-alarm screams, because I do not think that this is what your results show, due to the way you measured 'reaction time' and the choice provided to the participants (particular scream types).

Alternatively, you could conduct an additional experiment where participants are asked to listen to alarm versus non-alarm screams and to select simply 'alarm' versus 'non-alarm'. If my hypothesis is correct, I would expect similar/faster reaction time when hearing alarm screams compared to non-alarm screams, with still a high misclassification of non-alarm sounds as alarm.
[Response] Please see our previous response to the comment above. We also have to note that In the fMRI experiment (experiment 4) we indeed compared the broader categories of alarm and non-alarm screams without having the partisans to do a scream classification task. These brain response should reflect the inner reactions of the participants (the stress response), but still we do not find a better processing for alarm screams at least on a brain physiological level. So we are still confident that our data do show a less efficient processing of alarm compared to non-alarm screams.

Specific comments:
'neutral screams': since these */a/* sounds are not screams, I would avoid the terminology 'neutral screams' and call these sounds 'control' or simply 'neutral' sounds.
[Response] We completely understand the intention of the reviewers’ comment, and the appropriate term has to be chosen for these non-scream-like vocalizations. However, we think that the term “neutral screams” seems most appropriate in this context, as mentioned on p5: ”… referred to as “neutral screams” for the sake of terminological simplicity…”. These vocalizations were not “neutral” by a strong definition of neutral or control stimuli, because they were vocalized with the same vocal intensity and effort as generic screams, but largely missing the rough nature of original screams. Given this higher vocal intensity then normal neutral voices, we opted for the term “neutral screams”. We hope that we can convince the reviewer with our reasoning, and would very much like to keep the term “neutral screams”, which also makes the writing of the paper more consistent and coherent.

P5. ANOVAs. When conducting multiple anovas on the same data (e.g. 7 level and 3 level ANOVAs), was any correction applied?

[Response] We did apply a correction at this level testing, and this seems a rather unusual statistical procedure. Corrections are usually applied to multiple test at the same level of comparisons (i.e. posthoc test for comparing pairwise levels within a factor), or when performing comparisons across several conditions of equal experimental level (e.g. what we did on p9 when performing multiple t-test on the same level). Furthermore, during the fMRI analysis, corrections are applied within a certain contrast (i.e. correcting for many t-test across all voxels), but no corrections are applied across the total number of contrasts performed (on the same set of voxels); all fMRI contrasts are performed at the same alpha level. This is why we did not apply corrections across multiple ANOVAs, since they largely differed in their factorial complexity, and introducing corrections here would have meant correcting across factors (and interaction) with different levels of statistical complexity.

P6. In addition to this classification of non-scream sounds by a model trained on scream sounds, it would be useful to see how screams vs non-screams can be correctly classified (in these two categories) based on a model trained on the whole dataset.
[Response] This is an interesting suggestion by the reviewer, but we feel it is right now beyond the scope of the current manuscript. Here we tested how the expression of an emotion in one domain (e.g. screams) can be informative about an emotion in another domain (e.g. nonverbal expressions). This analysis already tests the scream/non-scream distinction at the level of discriminating emotions across domains (which is the focus of the current paper). The more general comparison as suggested by the reviewer mixes up different emotions at the major level, which we think is less interesting for the current paper. But as mentioned before, this is somehow already included in the analysis that we present in the manuscript.

P7-9. Throughout these results, the corresponding figures should be better referenced. They are sometimes missing.
[Response] We include now a better referencing to figures in the results section.

P7. The first sentence of this page ('expressing scream calls… senders') is awkward and would need to be revised (I guess you meant 'receivers' at the end)?
[Response] Yes, was changed accordingly.

P7. 'These 3 categories… than alarm screams (p=0.026).' might need revisions. According to the corresponding figure, the first 'faster' seems to be wrong. Should it not be 'slower' instead?
[Response] Yes, was changed accordingly.

P7. Last sentence starting by 'Thus, participants tend to…' (P8). This sentence is confusing, especially regarding 'perceive screams as 'alarming'… during misclassification of … alarm screams'. Alarm screams cannot be misclassified as alarming since they are actually alarming. I guess what you meant is that misclassified alarm screams tended to be classified as belonging to another of the alarm scream categories.
[Response] Yes, the sentence reads confusing, and the reviewer is right that the intended meaning was misclassification in another alarming scream category. We accordingly changes the sentence on p8: "Thus, participants tend to perceive screams as "alarming" when they misclassify scream calls, that is, they more likely choose one of the alarming scream types during misclassification. This was true during misclassifications of both non-alarm screams (i.e. classified in one the three alarm scream types) and especially of alarm screams (i.e. classified in one of the other two alarm scream types instead of the target alarm scream type) (Fig. 2A)".

P18. There seems to be some repetitions here. It reads as if the sounds were analysed twice by extracting 88 parameters using the openSMILE toolbox (acoustic analysis p17 and machine learning approach p18). Please clarify whether this is really the case.
[Response] The reviewer is right, this sounded like a repetition. The acoustic analysis and acoustic feature extraction however was only performed once. We now edited this section of the manuscript such that it reads to include one single acoustical analysis.

P19. Comparison of scream sounds and other standard nonverbal expression of emotions (e.g. joyful laughter, anger and growls): very few details are provided about how these sounds were recorded and who were the speakers. Were these sounds taken from an existing database? If not, how were they recorded, what was the age of the 16 speakers, and were some of them the same people who were producing the screams?
[Response] We added more information on the nonverbal expressions in the manuscript, p22: "(mean age 30.38 years, SD=7.07, age range 21-47; 10 of these 16 speakers also vocalized the scream calls used here)".

P20. How were the participants listening to the sounds? Through some headphones?
[Response] Yes, participants were wearing headphones. This is now included on p22: "Screams were presented at 70dB SPL using how quality headphones with a flat frequency profile (Sennheiser® HAD 280), and participants were asked to perform a 7AFC task according to the emotional valence of the screams ("neutral," "pleasure," "sad," "joy," "pain," "fear," "anger")".

P20. Reaction time. Was the reaction time calculated as the latency to click on one of the scream type options? This term could also be better defined the first time it is mentioned in the results (P7).
[Response] We now provide a definition of how we calculated response times and accuracy data. P23: "RTs were defined as the response latency from stimulus onset to the button press, and RTs were only calculated for correct trials. Accuracy was defined as pressing the one button that corresponded to the pre-assigned category of a scream (e.g. pressing the button for "fear" when a fear scream was presented)".

P21. It is not clear to me how the 2 scream presentation was done. Where the two screams played one after the other? Were the participants asked to choose a category after hearing two calls or after each call was played? After reading the text several times, I think I understand that they were played for example either a fear or sad scream several times in a row during a block and had to click right if they thought this was a fear call and left if they thought this was a sad scream, is that correct? This could be better explained, along with how the calculation of reaction time and accuracy was done.
[Response] Screams were presented as single trials in a random order, one block contained only two types of screams, and after each scream presentation participants had to press one of two buttons that belonged either to one or the other scream type.

P24: "Screams were presented as single trials in a random order, and after each scream participants performed a 2-alternative forced-choice (2AFC) task by pressing one button (left index finger) for one scream category (e.g. "anger") and another button (right index finger) for another scream category (e.g. "fear"). In each block, screams were presented in random order at 70dB SPL, with an ITI of 2250ms +/-250ms. Button and response option assignments were counterbalanced across blocks and participants.

Data analysis. For each of the 21 combinations of screams, we scored the mean RTs (i.e. response latency from stimulus onset to button press, only for correct trials) and accuracy data (i.e. correct trials were defined as button press that corresponded to the pre-assigned scream

type) for each of the 2 scream categories, as well as the mean difference in RTs and accuracy data between the 2 categories for each participant".

P22. What were the participants tested in the scanner told about the aim of the experiment?
[Response] Participants were told that the experiment is about the neural processing of different scream types. This is now highlighted on p25: "Participants were informed about the aim to investigate the neural dynamics of processing different types of screams, and they were informed to perform a task that is orthogonal to the emotional dimension of screams" and p23: "Participants performed a gender decision task, which is a task that is orthogonal to the relevant dimension of processing the different types of screams. This gender task has been used in previous studies and was established to be validly orthogonal to the emotion dimension of stimuli [1,8]".

Figure 2. It would be clearer if letters (a-h) are added next to each panel. This would allow a clearer description of the figure in the legend and would also be useful for the result section. In addition, the x-axis on the third panel on the first row (two-by-two comparisons) is very hard to read. Please space the categories out.
[Response] Was changed accordingly.

References. There are some repetitions in the references (e.g. number 1 is the same as number 20).
[Response] This is due to an awkward outcome of our reference manager software. We tried to clean up the reference list for these duplicates now.

Reviewer #3: PBIOLOGY-D-20-00286R1, Title: Neurocognitive processing efficiency for non-alarm rather than alarm signaling in human scream calls, Authors: Frühholz, S.; Dietziker, J.; Staib, M.; Trost, W.

Scream calls have long been assumed to serve an alarming purpose by signaling fear. In four experiments using perceptual and neuroimaging measures, this study found evidence for six generic and psycho-acoustically distinctive types of scream calls. Discrimination and recognition performance was worse for alarming screams compared to non-alarm screams. The neural signal and connectivity was minimal for alarming screams, at variance with a threat processing bias. Not only does this study highlight the diversity of different kinds of screams, it also indicates processing advantage for non-alarming and positive screams in communication.

Overall, this is a comprehensive and careful investigation of a topic relevant for a wider readership, including those interested in human voice perception and processing of affective vocalizations, as well as evolutionary aspects of human communication. Obvious strengths of the study include the use of various measures (acoustic, perceptual and neurophysiological), and a carefully selected and evaluated stimulus set of scream calls. The manuscript is well-written, methods and statistical analyses are sound and of high quality. The findings highlight the diversity of human scream calls and differences in their processing which challenge the traditional view that the brain has evolved to process alarming (negative) calls. This contributes a valuable new perspective on the communicative purpose of screams. I believe that the manuscript is already in excellent shape for publication in PLOS Biology.
[Response] We thank the reviewer for this very positive feedback on our manuscript. We revised the manuscript according to the reviewer comments.

Minor comments
It would also be more informative to have effect sizes in the results section.
[Response] Effect size measures (eta squared) were now included were applicable and relevant.

General remark: p-values are usually reported starting with the decimal point (p < .001, rather than 0.001).
[Response] Looking at other papers published recently in PLOS Biology, it seems that "0.001" seems the correct format for reporting p-values in this journal.

p. 4: typo in "screams calls" (scream calls)
[Response] Was corrected.

p. 5: typo in "a scream calls" (scream call)
[Response] Was corrected.

p. 22: typo in "date processing" (data processing)
[Response] Was corrected.

Reviewer #4: [identifies himself as Harold Gouzoules]
This is a very interesting manuscript, which, in fact, parallels work that has been done and is ongoing in my lab. I resonate with a number of the conclusions drawn by Frühholz and colleagues, but I have a number of suggestions and concerns about the stimuli used and the broad conclusions offered.
[Response] We thank Harold Gouzoules very much for the very interesting and constructive comments on our manuscript. We of course know the scientific work of this reviewer very well, and we tried to include some suggested perspectives as mentioned in the comments in our manuscript. Many of the valuable comments really helped to improve the manuscript and to positon our data in the broader field of animal and human communication research.

The study's stimuli come from 12 volunteers asked to generate screams that they think would be associated with six different scenarios. Nothing is said about these participants (other their mean ages and that that they were healthy. Problematically, the authors assume that these 12 individuals have the acting talent to generate different screams as they were asked to do. Have the authors seen the Brian De Palma film Blowout (1981)? The plot centers on the post-production phase of a low-budget slasher film, and the sound technician, played by John Travolta, is told by his producer that he needs a more realistic-sounding scream, Thus the plot starts with a bad actress in the film who cannot produce a convincing fear scream!

While Engelberg & Gouzoules (2018) found that participants in their study could not distinguish acted from natural screams, all of their acted screams came from talented (in the sense that they were in TV or major movie productions) actors. Engelberg and Gouzoules note: "It is important to emphasise that the chance-level performances demonstrated here do not necessarily imply that acted and natural screams are identical in every respect, nor that screams from all actors are equally suitable for implementation in empirical research."
[Response] If screams are within the communication repertoire of every human, it should be that not only talented actors (NB: being an actor on TV might not necessarily imply that someone is talented) are able to produce valid vocalizations of screams, but every normal human being with precise instructions. We took care that scream vocalizations were produced at the intended level of screaming. Furthermore, give the acoustic similarities our screams to screams reported in other

papers, we are confident to have a recorded a scream database with our speakers that is of a valid nature. Furthermore, all scream vocalizations subjected to an evaluation procedure.

To fully quote the section from the Engelberg 2018 paper: “It is important to emphasise that the chance-level performances demonstrated here do not necessarily imply that acted and natural screams are identical in every respect, nor that screams from all actors are equally suitable for implementation in empirical research. Our goal was to determine whether it is physically possible for humans to produce credible screams on command. **That we demonstrated no perceptual differences between acted and natural exemplars implies that there is nothing that intrinsically prohibits the deliberate production of convincing facsimiles**.”

Within the field of voice signaling research there is a big discussion if studies should use more natural vocalizations (with the disadvantage of having sampling biases, noisy backgrounds, unequal quality between signal categories, etc.) or using acted and recorded voice signals in the lab. Only the latter allows a more precise sampling of vocalizations and database creations, precise vocalization instructions, clean recordings, and equal sampling of vocalizations across categories. Since in this study we were interested in conducting precise psychoacoustic experiments and also precise neuroimaging experiments (which can be easily affect by degraded stimulus quality), we opted for using acted scream vocalizations, given that there are perceptually very close to natural screams (see quote above).

The sample size of participants asked to perceptually access the scream stimuli is remarkable small (experiment 1: 23, experiemtn 2: 33). In our similar studies, we typically use a minimum of 100 participants. Schwartz et al. 2019 ("Was that a scream? …") used 181 participants.
[Response] The reviewer raises an important point here, and sample size is an important issue when conduction psychoacoustic experiments. We however feel that our sample size is appropriate to the scientific questions we ask:

(1) The question about sample size is always relative, and in general, large sample sizes can have disadvantages since in psychoacoustic experiments a large sample often and easily lead to an overpowered statistics.

(2) Compared to most of the Gouzoules we use many more trials per participant to be able provide a better estimate of single subject responses. In psychoacoustic studies and studies in experimental psychology, there are always two options to increase power, either increasing the sample size or increasing the trial number per participant. The psychoacoustic literature always recommends the second option, since estimating single subjects measures is always of bigger important for calculating a proper mean across participants.

(3) For all analyses we used a multi-level random effects statistical modeling of the data; RFX analysis take the sample size into account and estimate how much the sample data are transferable to the population. Given all this reasoning, and given that the RFX models revealed significant effects, we are more than confident that our sample size are appropriate for our scientific questions.

Frühholz et al. cite an ASA abstract (Schwartz JW, Engelberg JW, Gouzoules H. What is a scream? Acoustic characteristics of a human call type. J Acoust Soc Am. 2019;145: 1776-1776. doi:10.1121/1.5101500) when they should cite the journal article (Schwartz, J. W., Engelberg, J. W., & Gouzoules, H. (2019). Was that a scream? Listener agreement and major distinguishing acoustic features. Journal of Nonverbal Behavior. https://doi.org/10.1007/s1091 9-019-00325-y) which provides much more relevant detail and discusses matters such as the Arnal et al. claims about roughness as a defining characteristic of human screams.
[Response] Thanks for pointing to this. The citation was changed, and we know refer to the JNB paper. Concerning the issue of roughness, we feel that the Schwartz 2019 does not really contradict the Arnal 2015, although the Schwartz 2019 give more acoustic and perceptual details. We also include a short notion in the manuscript, p3: “Other studies confirmed that roughness,

amongst other important acoustic and perpetual features, is a defining perceptual feature of screams that enable listeners to classify vocalizations as screams [9]".

Humans alone among primates scream in positive contexts (although some nonhuman primates occasionally scream during sex); the screams of other OW primates, though derived in function relative to those of non-primate animals, still occur in negative contexts. The central conclusion of this ms is, or at least implies, that in humans positive screams are primary from a functional/communication standpoint. If true, this would represent quite an extreme evolutionary divergence from other primates. In particular, the claim that "the primate neural system is more prepared to decode non-alarming and positive information signaled in human scream calls" is very surprising given that nonhuman primates do not encode positive information in scream calls. For the reader to grasp the extraordinary nature of these conclusions, the primate scream literature should be more thoroughly reviewed and discussed in relation to the conclusions of the paper. Even if the conclusion about positive screams is softened, a deeper discussion of the primate literature is warranted; the functions of NH primate screams cannot be reduced to "some specific type of vocal alarm signals." Thus, I strongly suggest that the authors do not give enough recognition to the nonhuman primate scream literature which shows that these vocalizations function in more complex ways than to signal alarm. These screams are acoustically diverse both within and among species) and function to recruit support from allies, usually matrilineal kin. I have added selected references at the end of this review.

[Response] The reviewer is correct that we should discuss our findings more clearly in the context of the animal literature. By reviewing the animal literature, we had the impressions that non-human primates largely express agonistic/recruitment screams by lower ranking animals (i.e. support from allies); in these interaction it also seems that aggressive higher ranking animal screams (which does not function to recruit allies rather than to intimidate the other animal). In the literature also "SOS screams" that really function as alarm signals are reported.

P3: "In nonhuman primates [10–12] and other mammalian species [13], scream-like calls are frequently used as some specific type of alarm signal exclusively in negative contexts, such as in in-group social conflicts between rank different animals. Screams by lower ranking animals help to recruit support from allies [14,15], while higher ranking animals scream to intimidate the lower ranking [16]. Furthermore, "SOS screams" signal the presence of environmental threat (e.g. predators) [17,18]. Scream-like voice calls thus aim to trigger certain behavior in potential listeners, similar to other types of alarm calls that are commonly expressed in high-arousal states of fear [19] or aggressiveness [20]".

P16: "… such that the human neural system is more prepared to decode non-alarming and positive information signaled in human scream calls in a mostly bottom-up manner. These findings in humans are surprising and largely diverge from studies in non-human primates; for latter, the literature so far only reported scream calls being expressed in negative contexts. While screams can also be expressed in negative contexts in humans, humans seem to be the only species to express screams in non-alarming and especially in positive contexts".

We also edited the final concluding paragraph, p17: "Taken together, this overall pattern of psychoacoustic, decision-making, and neural results for alarm screams in humans seems rather unusual for sociobiological voice calls when reviewed in the broader animal communication field. Scream calls were previously assumed to be most crucial in signaling and accurate communication of alarm across a broad range of animal species [11,13,21], sometimes also including references to humans [22,23]. Scream calls in non-human primates and other animals have been so far been reported to be expressed and perceived exclusively in negative contexts. A different picture of scream calls seems to emerge when investigated in humans, such that human listeners overall respond more quickly, more accurately, and with higher neural sensitivity to non-alarm and positive scream calls, which seem to have a higher relevance in human sociobiological interactions [3–5]. There seem some exceptions from this overall pattern of

scream recognition in humans, but across many psychoacoustic, behavioral, perceptual, and neural effects quantified here, alarm screams often show less neurocognitive processing efficiency than non-alarm screams. Alarm cream categories only have some primacy during misclassification of other scream types, which might be a safety choice under conditions of decisional uncertainty. And this safety choice might be shared with other non-human species that use screams in their vocal repertoire".

-The decision to classify screams reflecting different emotions into the categories of alarm/non-alarm follows the framework established by Arnal et al., but is odd in the context of communication research. In particular, "alarm" traditionally connotes a specific kind of socioecological context (typically, the presence of a predator) to which an animal is responding, rather than the animal's emotional or motivational state per se. The decision to equate emotion categories with levels of alarm contrasts with other, more established frameworks for the categorization of emotion (e.g., the dimensional framework). This should be discussed.
[Response] The reviewer mentions an important point here. We agree that "alarm" is often based on socioecological contexts (e.g. predators), but we also think that only the context is not sufficient enough to define "alarm"; it seems that we need both the context (e.g. a leopard as predator) as well as the animal responding in a certain way to the context, such that the context has relevance for this specific animal (e,g, a monkey) (maybe not for other animals; e.g. an elephant). So to define "alarm" we cannot ignore the specific animal's motivational (emotional?) state. Furthermore, if we look at humans (which is the primary focus of our manuscript), almost all screams seem to be driven by emotional states elicited in certain contexts, and some of these contexts have an alarming nature for humans.

In our manuscript we take a semi-categorical emotion approach, by including emotional categories, but also including dimensional arousal ratings. The pure dimensional approach in emotion research is quite old (Russel's work from 1980), and more recent conceptual emotion approaches are often hybrid models between dimensional and categorical features. Based on this, we classified screams into certain scream types, but these screams were then rated in a dimensional approach both along their arousal level and their alarming level. We also have to note that screams were rated individually, not as being member of a certain scream type. After being rated individually, we can quantify the mean alarm rating for scream type.

Screams are expressed by the sender to be registered by other animals/individuals, and listeners potentially assess screams both along their alarm level (how urgent do I need to respond), and along their affective quality (is it pain or anger?).

We included a discussion about this issue now in the manuscript, p5: "Although the term "alarm" often refers to certain sociobiological contexts and events that elicit screams especially in animal settings, the scream can be regarded as the senders' expression of the alarming significance of the context or event. Listeners than rate the alarming level of these screams as part of their decision how urgent one needs to respond. We quantified these listeners alarm rating individually for each scream and on a dimensional basis (i.e. intendent of the knowledge to which category the scream belonged to)."

Finally, the alarm level was entered as a parametric regressor independent of the emotion categories for the analysis of the fMRI data (Fig. 4). The resulting data are largely resembling the data obtained from making categorical comparison between alarm and non-alarm screams. This nicely shows that the dimensional and the categorical approach go nicely hand in hand.

Specific comments:

* Page 3, line 9: "scream-like calls are most frequently used as… vocal alarm signals" - I wouldn't call them alarm signals since alarm calls are a very different call class in the animal communication literature.

[Response] We completely understand the reviewer's point that one should be careful not to mix up the modalities of scream or scream-like calls (e.g. agonistic screams, recruitments screams, distress screams, "SOS screams" etc.) and alarm calls. However, we think that scream calls can have different in-group and out-group functions in primates (maybe even in mammals and vertebrates) that can be described as "alarm signals" (NB: not to mix up with alarm calls). The reviewer mentions in a comment above that one function is to "recruit support from allies"; this would not be effective if the screams would not include some alarming quality (support needs to arrive immediately!) and some emotional state in the screaming animal. Furthermore, for in-group social conflicts the literature also reports screams be the higher ranking animal [16], which obviously has the function to intimidate the lower-ranking animal. These aggressor screams seem also to imply some form of alarm signal (maybe scare the lower ranking animal), and seem also driven by emotional states of the aggressor.

So overall, we think that the term "alarm signal" seem not inappropriate, give also that some screams generically function as alarm signals ("SOS screams") to conspecifics [17,18]. Taking all these considerations into account, we edited the respective section in the manuscript to introduce these distinctions, p3: "In nonhuman primates [10–12] and other mammalian species [13], scream-like calls are frequently used as some specific type of alarm signal exclusively in negative contexts, such as in in-group social conflicts between rank different animals. Screams by lower ranking animals help to recruit support from allies [14,15], while higher ranking animals scream to intimidate the lower ranking [16]. Furthermore, "SOS screams" signal the presence of environmental threat (e.g. predators) [17,18]. Scream-like voice calls thus aim to trigger certain behavior in potential listeners, similar to other types of alarm calls that are commonly expressed in high-arousal states of fear [19] or aggressiveness [20]".

* Page 3: "expressed in less intense nonverbal vocal emotions such as sadness, joy, and pleasure" - What is meant by pleasure? Sensual pleasure?
[Response] Yes, we meant sensual pleasure. This was now clarified in the manuscript, p4: "However, humans scream not only when they are fearful and aggressive, but also when they experience other affective states, similar to a variety of emotions that are more commonly expressed in less intense nonverbal vocal emotions [24], such as sadness, joy, and sensual pleasure"

* Page 5: if the authors used ratings of alarming quality, and they are insistent on the alarm vs. non-alarm taxonomy, why not group screams based on their ratings (high alarm ratings vs. low alarm ratings), rather than grouping all pleasure, sad, and joy as non-alarm, and all pain, anger, fear as alarm? Perhaps, for example, some joy screams were rated as more alarming than some anger screams? What inherently makes "anger" more alarming than "joy"?
[Response] This is an important point, and our reasoning is largely based on sample statistics. If the question is that one category of screams has a higher mean level of alarm ratings than another category of screams, one needs to compare the distributions between both categories. If a significance test is significance, it means that the distributions are different (which does not exclude the fact that the distributions can overlap), and that most members of one category have larger/smaller values than members of the other category. If the distribution for alarm ratings of anger screams is significantly higher/different than for joy screams, one would say that based on these statistics anger is "inherently" more alarming than joy. We think based on this reasoning we have valid evidence for separating scream categories based on their group alarm ratings.

Furthermore, based on the suggestion by the reviewer, we included one analysis approach for the fMRI data, where we ranked screams solely based on their alarm ratings and independent of the category, and found that the alarm rating correlated negatively with neural effects in several brain areas (Fig. 4); these neural effects largely replicate the findings for categorically comparing non-alarm > alarm screams (Fig. 3b).

* Page 6: Unclear to me that their definition of roughness is the same as Arnal et al.'s. I believe roughness is ultimately a perceptual attribute (that correlates with amplitude modulations), but this study never had listeners rate roughness.
[Response] We used the same procedure to determine the roughness as in the Arnal et al paper; this is now mentioned in the manuscript (p6). Roughness so far is technically defined as an acoustic features based on the temporal and spectral modulation rates in vocalizations that largely corresponds to certain perceptual impression when listening to screams (although the precise acoustic-to-perception relationship still needs to be defined). Roughness is probably not only a perceptual feature, but it seems most likely associated to spectro-temporal modulations rates. In our study roughness correlated with the alarm rating, so there is a correspondence between the acoustic and the perception effects.

* Page 7: "selection of 84 of the original 420 scream calls" - more information about the selection process would be helpful.
[Response] This selection procedure is in detail described in the methods section (Experiment 2 – Perceptual categorization of scream calls), including all selection criteria based on statistical testing, p22: “From the acoustic scream recordings of experiment 1, we selected 84 screams (3 male, 3 female speakers) with 2 instances of screams per category. Stimuli were selected from the results of the perceptual assessment of screams in experiment 1, such that no significant differences in the recognition rate across scream types ($F_{1,6}$=1.895, p=0.085) were found for this selection. Mean arousal level differed across all 7 scream types ($F_{1,6}$=51.065, p<0.001), across the 6 generic screams ($F_{1,5}$=11.647, p<0.001), and between neutral, alarm, and non-alarm screams ($F_{1,2}$=77.645, p<0.001)”.

* Page 7: Was reaction time calculated for all trials or only correct ones? Were extreme outliers excluded?
[Response] Yes, RTs were only calculated on correct trials. Outlier trials (>3SD per each participant) were excluded if in the data.

* Page 8: Is d' appropriate given multiple response categories? It seems like d' is usually used for two-category judgments to show the discriminability of two groups.
[Response] In general, d primes can be used for any number of response options as long there is no bias towards a category.

* Page 8: I am concerned about the sample size for, in particular, experiment 3 given the many different decision tasks that are compared
[Response] See our response to the comment above. It would have been interesting to have more details in this reviewer comment, and to understand in which direction the reviewer is concerned. Is it a statistical concern? In a response above, we outlined the notion that we used many trials per participants, which allows a proper estimation of single subject measures. Laos, we used FDR correction for the different comparisons done. Give these experimental and statistical conditions, we are confident that our data and results are statistically robust.

* Page 9: "Again, we have to note all types of screams were acoustically very distinct from each other at a comparable level, such that discrimination impairments… were unlikely driven by potential acoustic similarities." - This needs more documentation. I am surprised and need more convincing that all scream types are equally acoustically distinct and that acoustics have little to do with discrimination impairments. (Actually, their acoustic classifier's accuracy ranges from 65.8% for sad to 89.7% for joy - arguable that they are distinct at a "comparable" level.

[Response] The reviewer mentions an important point here, since the word “comparable” might be misleading here. The reviewer is right that the classifiers accuracy level with different for the different types of screams. With the word “comparable” we wanted to highlight the fact that all accuracies levels of the classifier were very high above chance level (14%); even for the lowest performance (65.8%) it was >50% above chance level, which is, in terms of general machine leaning classifier performance, a very high performance level. We edited the respective section in the manuscript to be clearer here, see below.

P9: “We have to note that this pattern of results is unlikely to be driven by the acoustic (dis-)similarity of some the 6 generic screams, as the machine-learning approach in the previous paragraph has shown a relative large acoustic distance (i.e. high above chance discrimination of the machine classifier; Fig. 1d) between all types of screams”

p11: “Again, we have to note that all types of screams were acoustically very distinct from each other given the high above chance level classification according to the outcomes from the machine learning approach (Fig. 1d), such that discrimination impairments between screams were unlikely driven by potential acoustic similarities”.

* Pages 14-15 - The concluding paragraph makes quite bold claims and should either be tempered and/or have better grounding in the animal literature.

[Response] We revised the concluding paragraph along the suggestions by the reviewer, p17: “Taken together, this overall pattern of psychoacoustic, decision-making, and neural results for alarm screams in humans seems rather unusual for sociobiological voice calls when reviewed in the broader animal communication field. Scream calls were previously assumed to be most crucial in signaling and accurate communication of alarm across a broad range of animal species [11,13,21], sometimes also including references to humans [22,23]. Scream calls in non-human primates and other animals have been so far been reported to be expressed and perceived exclusively in negative contexts. A different picture of scream calls seems to emerge when investigated in humans, such that human listeners overall respond more quickly, more accurately, and with higher neural sensitivity to non-alarm and positive scream calls, which seem to have a higher relevance in human sociobiological interactions [3–5]. There seem some exceptions from this overall pattern of scream recognition in humans, but across many psychoacoustic, behavioral, perceptual, and neural effects quantified here, alarm screams often show less neurocognitive processing efficiency than non-alarm screams. Alarm cream categories only have some primacy during misclassification of other scream types, which might be a safety choice under conditions of decisional uncertainty. And this safety choice might be shared with other non-human species that use screams in their vocal repertoire”.

* Page 15 - "These types of screams were chosen to comprehensively cover all possible screams that humans vocalize…" - "Comprehensive" seems a bold word here. What makes it comprehensive? How would the authors know? I can think of other contexts!

[Response] The background is that we thought a lot about contexts and emotional conditions that could potentially elicit scream like vocalizations in humans. We also checked the literature to check for any ideas about possible emotional conditions that elicit screams. Although we agree with the reviewer that there are many more contexts (we would have really liked if the reviewer would have listed some more example contexts), it seems that many of these contexts elicit kind of screams, at least in humans. Thus, the contexts might be diverse, but the screams elicited might be based on the types of screams we describe here. To downtone the section in the manuscript, we know use the word “broad” or “broader” to describe the fact that we included a variety of scream types in our studies.

P4: “This broader taxonomy of screams is proposed based on a survey of many daily natural, social, and cultural manifestations of human screams, and the general diversity of human affective vocalizations [25] and especially of nonverbal expression of emotions [24]”.

* Page 16 - (After discussing the standardization of scream duration + amplitude): "..this procedure ensured that all following analyses and perceptual experiments are not confounded by basic and rather unimportant acoustic features of these screams." I understand why they standardized, but I don't think it's absolutely clear that duration and amplitude are likely to be "unimportant"; I wouldn't use that language.
[Response] We changed this section. The term “unimportant” might be indeed misleading here and was deleted. The background for the standardization procedure is that in precise psychoacoustic and neuroimaging experiment, one has to carefully match stimuli for features that might induce significant differences themselves. We know that stimulus duration and stimulus intensity can significantly drive brain activity (especially in auditory cortex), and in order to avoid such confounding effects we opted for the standardization (also for the behavioral experiments) being aware that some stimuli might have sounded less “natural”. But we think the precision in the experimental setup outweighs the effects introduced by standardization.

* Page 17 - "Valence was quantified by a 7-alternative forced-choice classification ('neutral,' 'pleasure,' 'sad,', 'joy,' 'pain,' 'fear,' 'anger')." In the emotion literature valence is usually conceived of as a dimension from negative to positive. I wouldn't really call this a valence judgment. Is the difference between "sad" and "fear" valence based?
[Response] The reviewer mentions an important point, but valence also has a broader meaning emotion literature as being limited to one dimension (see [26]). To avoid confusion here, we now choose the term “emotional category” judgement.

* Page 7 - "We selected 84 screams" - again, selected on what basis?
[Response] This is documented on p22: “From the acoustic scream recordings of experiment 1, we selected 84 screams (3 male, 3 female speakers) with 2 instances of screams per category. Stimuli were selected from the results of the perceptual assessment of screams in experiment 1, such that no significant differences in the recognition rate across scream types ($F_{1,6}$=1.895, p=0.085) were found for this selection. Mean arousal level differed across all 7 scream types ($F_{1,6}$=51.065, p<0.001), across the 6 generic screams ($F_{1,5}$=11.647, p<0.001), and between neutral, alarm, and non-alarm screams ($F_{1,2}$=77.645, p<0.001)”.

* Page 20 - "maximum response time window of 3000 ms" - so subjects had 3 seconds to respond. What happened if they did not respond in that window? Did this lead to missing data? How much?
[Response] Yes, no response within 3000ms were marked as missing data. Across conditions, we had an average of 6.53% trials classified as missed. This is now added to the manuscript, p23: “Screams were presented in random order at 70dB SPL, with a maximum response time window of 3000ms; trials with no response in this time window were classified as misses trials (6.53% of all trials)”.

* Page 22 - In the MRI participants were told to judge speaker sex as a "simple" task to "keep participants attentive." Do we know this was simple for them? How did they do?
[Response] We included the behavioral data of the gender task now in the manuscript, see Fig. S2. The statistical analysis were included on p11: “Reactions times did not differ between all 7 scream types ($F_{6,174}$=2.371, p=0.094) nor between the 3 major categories of neutral, non-alarm, and alarm screams ($F_{2,58}$=0.756, p=0.428). Error rate was different between all 7 scream types ($F_{6,174}$=7.280, p<0.001, η^2=0.10), but not between the 3 major categories ($F_{2,58}$=2.177, p=0.132) (Fig. S2)”.

Although participants made errors during the task, it seemed simple especially from the viewpoint of the task instructions and the number of response options. To be clearer here we replace the word “simple” by “basic”.

* Figure 2A. The reaction times between alarming and non-alarming really don't look all that different - seems like a faster reaction to joy is driving it; the other non-alarm ones don't look significantly different from alarm.
[Response] This is a good point, and RTs to joy are really fast. First, the statistical analysis leads to the results reported in the manuscript, which we cannot ignore. However, it also seems that response to pleasure are somehow faster to some alarm screams. Give this, we included a notion in the manuscript to highlight this issue, p8: “These 3 categories differed in reaction times (1w-ANOVA, 3 levels; $F_{2,64}$=27.636, p<0.001, η^2=0.20), with non-alarm (p<0.001) and alarm screams (p<0.001) having slower mean reaction times than those for neutral screams, and non-alarm screams being classified faster than alarm screams (p=0.005) (Fig. 2a). The latter difference however seems strongly driven by fast response to joy screams, and partly also to pleasure screams compared the other three alarm screams”.

* Figure S1. Examining the accuracy rates for all 420 screams, the % accurate for joy really doesn't look all that different from pain or fear. The bigger difference seems to be in false alarm rates, which are higher for pain, fear, and anger. How does this comport with the authors' conclusion that "non-alarm and positive scream calls… seem to have a higher relevance in human sociobiological interactions"? A tendency to misattribute screams as pain, fear, or anger would suggest that those ("alarming") screams have primacy to the point that even many non-alarm screams are interpreted as alarm.
[Response] This is also a good point, and we should differentiate these two important observations: (1) across many measure, non-alarm screams have better processing efficiency and (2) during misclassifications, the alarm scream categories are more frequently chosen. These are two important and partly independent observations, and we separate these two points at many points in the manuscript, e.g.:
P8: “Thus, participants tend to perceive screams as “alarming” when they misclassify scream calls, that is, they more likely choose one of the alarming scream types during misclassification. This was true during misclassifications of both non-alarm screams (i.e. classified in one the three alarm scream types) and especially of alarm screams (i.e. classified in one of the other two alarm scream types instead of the target alarm scream type) (Fig. 2d)”.
P15: “The only priority that alarming scream categories received was their frequent use during misclassifications of other screams in a multi-option classification task in experiment 2. This might resemble a natural threat perception bias that seems to be a cost-benefit efficient solution when balancing response options against potential sources of (non-)threat, especially under conditions of uncertainty [27]”.
P17: “… non-alarm and positive scream calls, which seem to have a higher relevance in human sociobiological interactions [3–5]. There seem some exceptions from this overall pattern of scream recognition in humans, but across many psychoacoustic, behavioral, perceptual, and neural effects quantified here, alarm screams often show less neurocognitive processing efficiency than non-alarm screams. Alarm cream categories only have some primacy during misclassification of other scream types, which might be a safety choice under conditions of decisional uncertainty”.

Additional relevant literature for the authors and editor to consider:
Schwartz, J. W., Engelberg, J. W., & Gouzoules, H. (2019). Was that a scream? Listener agreement and major distinguishing acoustic features. Journal of Nonverbal Behavior. https://doi.org/10.1007/s1091 9-019-00325-y
Gouzoules, H. (2005). Monkeying around with symbolism: Are vocalizations simple symbols...or more like cymbals? In L. Namy (Ed.), Symbol use and symbolic representation: Developmental

and comparative perspectives. (pp. 245-265). Mahwah, NJ: Lawrence Erlbaum Associates Publishers.
Gouzoules, H., & Gouzoules, S. (2000). Agonistic screams differ among four species of macaques: The significance of motivation-structural rules. Animal Behaviour, 59(3), 501-512. https://doi.org/10.1006/anbe.1999.1318
Gouzoules, H., & Gouzoules, S. (2006). The conundrum of communication. In C. Campbell, A. Fuentes, K. MacKinnon, M. Panger, & S. Bearder (Eds.), Primates in perspective (pp. 621-635). Oxford: Oxford University Press].
Gouzoules, H., Gouzoules, S., & Marler, P. (1985). External reference and affective signaling in mammalian vocal communication. In G. Zivin (Ed.), The development of expressive behavior: Biology environment interactions (pp. 77-101). New York: Academic Press.
Gouzoules, H., Gouzoules, S. and Marler, P. (1986), Vocal communication: A vehicle for the study of social relationships. In: The Cayo Santiago Macaques, R.G. Rawlins and M.J. Kessler, eds., pp. 111-129. State University of New York Press: Albany.
Gouzoules, H., Gouzoules, S., & Tomaszycki, M. (1998). Agonistic screams and the classification of dominance relationships: Are monkeys fuzzy logicians? Animal Behaviour, 55(1), 51-60.
[Response] Thanks for these papers suggestions. Although this list seems limited towards the reviewers' own work, we especially appreciate the suggestions of more recent work and included some more references in the manuscripts.

References

1. Grandjean D, Sander D, Pourtois G, Schwartz S, Seghier ML, Scherer KR, et al. The voices of wrath: brain responses to angry prosody in meaningless speech. Nat Neurosci. 2005/01/25. 2005;8: 145–6. doi:10.1038/nn1392
2. Frühholz S, Hofstetter C, Cristinzio C, Saj A, Seeck M, Vuilleumier P, et al. Asymmetrical effects of unilateral right or left amygdala damage on auditory cortical processing of vocal emotions. Proc Natl Acad Sci U S A. 2015;112. doi:10.1073/pnas.1411315112
3. Ethofer T, Wiethoff S, Anders S, Kreifelts B, Grodd W, Wildgruber D. The voices of seduction: Cross-gender effects in processing of erotic prosody. Soc Cogn Affect Neurosci. 2008/11/06. 2007;2: 334–337. doi:10.1093/scan/nsm028
4. Charlton BD, Reby D. The evolution of acoustic size exaggeration in terrestrial mammals. Nat Commun. 2016;7. doi:10.1038/ncomms12739
5. Johnstone T, van Reekum CM, Oakes TR, Davidson RJ. The voice of emotion: an FMRI study of neural responses to angry and happy vocal expressions. Soc Cogn Affect Neurosci. 2007/07/04. 2006;1: 242–249. doi:10.1093/scan/nsl027
6. Stephan KE, Penny WD, Moran RJ, den Ouden HEM, Daunizeau J, Friston KJ. Ten simple rules for dynamic causal modeling. Neuroimage. 2009/11/17. 2010;49: 3099–3109. doi:10.1016/j.neuroimage.2009.11.015
7. Kumar S, Kriegstein K von, Friston K, Griffiths TD. Features versus feelings: Dissociable representations of the acoustic features and valence of aversive sounds. J Neurosci. 2012;32: 14184–14192. doi:10.1523/JNEUROSCI.1759-12.2012
8. Frühholz S, Hofstetter C, Cristinzio C, Saj A, Seeck M, Vuilleumier P, et al. Asymmetrical effects of unilateral right or left amygdala damage on auditory cortical processing of vocal

emotions. Proc Natl Acad Sci U S A. 2015;112: 1–6. doi:10.1073/pnas.1411315112

9. Schwartz JW, Engelberg JWM, Gouzoules H. Was That a Scream? Listener Agreement and Major Distinguishing Acoustic Features. J Nonverbal Behav. 2019. doi:10.1007/s10919-019-00325-y

10. Fichtel C, Perry S, Gros-Louis J. Alarm calls of white-faced capuchin monkeys: An acoustic analysis. Anim Behav. 2005;70: 165–176. doi:10.1016/j.anbehav.2004.09.020

11. Gouzoules S, Gouzoules H, Marler P. Rhesus monkey (Macaca mulatta) screams: Representational signalling in the recruitment of agonistic aid. Anim Behav. 1984. doi:10.1016/S0003-3472(84)80336-X

12. Le Prell CG, Hauser MD, Moody DB. Discrete or graded variation within rhesus monkey screams? Psychophysical experiments on classification. Anim Behav. 2002. doi:10.1006/anbe.2001.1888

13. Manser MB. The acoustic structure of suricates' alarm calls varies with predator type and the level of response urgency. Proc R Soc B Biol Sci. 2001;268: 2315–2324. doi:10.1098/rspb.2001.1773

14. Gouzoules H, Gouzoules S, Tomaszycki M. Agonistic screams and the classification of dominance relationships: Are monkeys fuzzy logicians? Anim Behav. 1998;55: 51–60. doi:10.1006/anbe.1997.0583

15. Gouzoules H, Gouzoules S. Agonistic screams differ among four species of macaques: The significance of motivation-structural rules. Anim Behav. 2000;59: 501–512. doi:10.1006/anbe.1999.1318

16. Mercier S, Déaux EC, van de Waal E, Bono AEJ, Zuberbühler K. Correlates of social role and conflict severity in wild vervet monkey agonistic screams. PLoS One. 2019;14. doi:10.1371/journal.pone.0214640

17. Zuberbühler K. Causal knowledge of predators' behaviour in wild Diana monkeys. Anim Behav. 2000;59: 209–220. doi:10.1006/anbe.1999.1296

18. Hopkins R, Folt B. Screaming Calls of Leptodactylus savagei (Smoky Jungle Frog) Function as an Alarm for Conspecifics. J Herpetol. 2019;53: 154. doi:10.1670/18-083

19. Blumstein DT, Récapet C. The sound of arousal: The addition of novel non-linearities increases responsiveness in marmot alarm calls. Ethology. 2009;115: 1074–1081. doi:10.1111/j.1439-0310.2009.01691.x

20. Raine J, Pisanski K, Oleszkiewicz A, Simner J, Reby D. Human Listeners Can Accurately Judge Strength and Height Relative to Self from Aggressive Roars and Speech. iScience. 2018;4: 273–280. doi:10.1016/j.isci.2018.05.002

21. Seyfarth R, Cheney D, Marler P. Monkey responses to three different alarm calls: evidence of predator classification and semantic communication. Science (80-). 1980;210: 801–803. doi:10.1126/science.7433999

22. Belin P, Zatorre RJ. Neurobiology: Sounding the Alarm. Current Biology. 2015. pp. R805–R806. doi:10.1016/j.cub.2015.07.027

23. Arnal LH, Flinker A, Kleinschmidt A, Giraud AL, Poeppel D. Human Screams Occupy a Privileged Niche in the Communication Soundscape. Curr Biol. 2015;25: 2051–2056.

doi:10.1016/j.cub.2015.06.043

24. Anikin A, Persson T. Nonlinguistic vocalizations from online amateur videos for emotion research: A validated corpus. Behav Res Methods. 2017;49: 758–771. doi:10.3758/s13428-016-0736-y

25. Frühholz S, Trost W, Kotz SA. The sound of emotions-Towards a unifying neural network perspective of affective sound processing. Neurosci Biobehav Rev. 2016;68: 1–15. doi:10.1016/j.neubiorev.2016.05.002

26. Shuman V, Sander D, Scherer KR. Levels of valence. Front Psychol. 2013;4. doi:10.3389/fpsyg.2013.00261

27. Bach DR, Dolan RJ. Knowing how much you don't know: A neural organization of uncertainty estimates. Nature Reviews Neuroscience. 2012. pp. 572–586. doi:10.1038/nrn3289
