# Supplementary material for: Neurocognitive processing efficiency for discriminating human non-alarm rather than alarm scream calls
Source: PLoS Biol. 2021 Apr 13;19(4):e3000751. doi: 10.1371/journal.pbio.3000751 (PMC8043411; doi:10.1371/journal.pbio.3000751)
Supplement: Attachment — Submitted filename: SFruhholz_reponse_to_reviewers.pdf [file pbio.3000751.s015.pdf]

Reponses to comments by the reviewers
PBIOLOGY-D-20-00286R1

REVIEWERS' COMMENTS:

Reviewer #1:

The authors chose to rebuttal the arguments of three of the four reviewers but unfortunately did not provide the requested additional analyses that could have helped to support the authors' arguments. I appreciate the authors arguments.

Response: We thank the reviewer again for these very constructive comments on our revised manuscript. We took the reviewer's suggestions very seriously, also including now new data from a replication of experiment 2 that show a large reproducibility of the data using a different selection of screams. The latter we think was also mentioned by reviewer #4.

We give a detailed response to the reviewer comments in the specific comments listed below. We took into account all the reviewer's comments, and now provide all additional information as requested by reviewer #1.

However, I would like to reemphasize the main problem that reviewer 2 and myself identified: Based on the currently presented data, the authors cannot state that there is a superior processing efficiency for non-alarm vs. alarm screams in general. This efficiency can only be stated for the task under investigation. Other tasks on the identical stimuli may reveal other results. E.g., the authors now provide the RT for alarm ratings that unsurprisingly do not show a superior processing efficiency for non-alarm calls when judging the alarm quality. This information should be included and discussed in the manuscript.

Response: The reviewer mentions an important point that performance data need to be viewed within the specific task investigated. Indeed, the RT data for the alarm rating did not show a difference between cream types. But we also have to note here, that the alarm rating was not a speeded reaction time task and it included the position of a slider of the screen for the participants. First, this task settings thus is not appropriate for quantifying the speed of classification, and participants were also not instructed to give speeded ratings. Second, as can be seen from the RT plot (see below), the mean reaction time was somehow located around ~3.5 seconds, which is almost the double amount of response compared to the speeded classification experiments (experiments 2 and 3). We assume that is very unlikely to find differences in RT at a response level between 3-4s.

Comparing the data from the evaluation and rating experiments (i.e. that were performed in a self-paced and non-speeded manner) with the data from the speeded classification experiments (experiments 2 and 3) it is clear that performance differences between different types of screams only show up in the speeded classification tasks. We again have to note that the evaluation and rating task were not performed with settings (sliders, pressing 1 out of ten buttons) that allowed speeded behavioral responses, and a response time level of 3-4s is also unlikely to reveal response time differences.

In terms of clarity, we now included statements in the manuscript that highlight the task dependency of the effects found across the different experiments.

P6: "In an additional analysis, we tested for response time difference for the alarm ratings (Fig. 2b) and found no difference across all 7 types of screams ($F_{6,132}$=2.192, p=0.098, η^2=0.01) nor between the three major categories ($F_{2,44}$=3.015, p=0.084, η^2=0.01). Thus, these self-paced alarm ratings were performed with a similar response latency across scream types, but with overall relatively slow responses (>3s) compared to the speeded classification tasks as we report below".

P8: "Having shown in experiment 1 that human screams are not limited to alarming screams signaling threat, but rather show an acoustic diversity of at least 6 different types of screams, we investigated in experiment 2 how accurately human listeners can perceptually discriminate and classify these different scream types in a speeded classification task".

P17: "Only in the speeded classification task, but not in the self-paced rating task that included making ticks on a visual analog scale on the screen, we found indication for some lower processing efficiency of alarm compared non-alarm screams. This indicates that this lower processing efficiency for alarm screams only occurs when humans have to make fast judgments on the scream signals perceived, and these fast judgments seem critical in some contexts".

P18: "This additionally suggests that alarm screams surprisingly need more processing effort during simple scream discrimination with only two choice options in a speeded discrimination task, and that rather non-alarm and positive screams are more efficiently processed during this perceptual discrimination of two types of screams".

P21: "Second, we found indications of some lower processing efficiency for alarm compared to non-alarm screams especially in speeded classification tasks including complex 7-alternative-forced choice options tasks as well as in a simpler two-option discrimination task. No such differences were found in a self-paced and non-speeded rating task that asked humans to rate screams along their alarm level. The lower processing efficiency for alarm screams thus seem task-dependent and seems to be observed in contexts that require speeded decisions".

Which unit is on the y-axis of the illustration in the response to reviewers; is it seconds?

Response: In our previous response to the comments of reviewer #1 we included two plots for reaction times. I copy these plots here again, and confirm that the y-axis is in the units of seconds

This is the first instance where we reported the RT distribution plot "Reaction times for alarm ratings did not show differences between neutral, non-alarm, and alarm screams ($F_{2,44}$=3.015, p=0.084); see below"

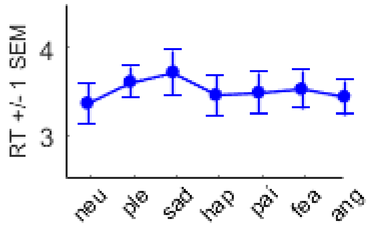

Reaction time plot with another scaling of the y-axis shows the error bars:

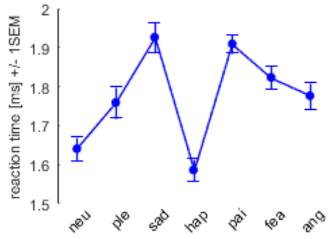

I strongly recommend discussing the significance of the current findings and relate these findings to previous reports in the literature showing, for example, a higher processing efficiency in terms of RT for alarm compared to non-alarm signals when judging the alarming quality.

Response: The reviewer mentions an important point, and such a discussion is of course very much warranted. However, one limitation here is that there is almost no research existing about the processing

efficiency of alarm signals directly. Instead, there is a lot of research on how hearing alarm signals can facilitate the performance on other tasks (i.e. target detection or object classifications cued by the alarm signal). We summarized this line of research now in the introduction, p3:

“Previous research on alarm signal perception indicated that alarm signals induce strong physiological and alertness reaction in listeners [1,2] that facilitates the detection of other visual and auditory targets [3,4], especially with increasing of levels of urgency perceived in the primary alarm signals [3,4]. However, there is research missing that quantifies the response time and the accuracy of processing the alarm signals itself besides the effects on the detection of secondary signals. Investigating the response speed and accuracy in the perception of screams as a unique type of alarm signals might be an ideal case for obtaining data on the processing efficiency of alarm signals themselves”.

Another major point that still lacks attention is that the selection procedure has not been sufficiently justified. The authors argue that the selection was performed such that the recognition rates did not differ significantly. However, in the other sample of 33 participants, the authors elaborate on exactly those differences. I am afraid I do not understand the logic behind this approach. It would help if the authors clarified this point and show that the results in experiment 2 are robust against replacement of the selected stimuli by other stimuli which also conform to the selection criteria.

Response: The reviewer mentions an important point here, which we want to further classify. The 84 scream stimuli that we selected out of the total 420 screams were selected such that there was no difference in the base recognition rate between the scream categories. The basis for this selection criteria was the data the ewe obtained from an evaluation of the stimuli by an independent sample as shown in Fig. S1. And as mentioned on p20 of the manuscript. The evaluation was done by an independent sample of n=26 participants in a non-speeded and self-paced manner, which we now highlight on p25:

“Both the alarm rating and the perceptual assessment acquisition were performed in a self-paced manner, such that participants could take their own time to perform the ratings; the next trial only started after the participants finished the ratings on the previous trial”.

The response times for assessing the emotional category during this evaluation experiment was ~3.5seconds as shown in the first RT plot above.

Unlike during the evaluation experiment, in the classification task described in experiment 2 participants were asked to give speeded responses in a maximum time window of 3s, described on p28:

“After a response option was chosen, the response screen disappeared and the next scream was presented after an inter-trial interval (ITI) of 1750 +/- 250ms. Participants could give their response in 3s time window; if no response was given within these 3s, the experiment continued with the next trial”.

This experimental design resulted in RT that were in the range of 1.6-1.9s, such that these responses were much faster compared to the evaluation experiment. In case participants are forced to give more speeded responses they usually tend to (differential) errors. To ensure that error rates during experiment 2 were not biased by different recognition rates between the selected stimuli, we choose to select those stimuli to have an equal base recognition rate. This seems also a valid procedure that is adopted by many other studies in the field.

P8: “We therefore presented a selection of 84 of the original 420 scream calls (see methods). All selected screams had an equal base recognition rate to ensure that different accuracy rates for different scream types in this experiment were not biased by a different base recognition rate of the selected stimuli. These selected screams were presented to another sample of human listeners (n=33) and asked them to classify the screams into 7 categories that referred to the 6 scream types and the neutral vocalizations (Fig. 3)”.

The reference to Figure S1 is missing from the appropriate text passage, the stats for the n=26 group analyses similarly.

Response: The reference to Fig. S1 was now included in the manuscript (p4). The general statistical analysis for the n=26 sample was now included below Fig. S1.

In the same line, the authors presented a good rationale for the standardization procedure but did not study whether this procedure affected the alarm vs. non-alarm screams and the scream types differentially. Even if it did, I believe that there could be something to learn about scream processing. If adjusting loudness affects alarm vs. non-alarm scream processing differently, this would identify loudness as a relevant (co-)factor in scream recognition.

Response: The rationale for the standardization of the screams sounds that were selected for the experiment is described in the manuscript on p24:

“The final selected screams were cropped to a fixed duration of 800ms, standardized to an identical RMS across screams corresponding to 70dB SPL, and faded-in and faded-out by a 15ms intensity ramp at the beginning and end of each scream. The final sample included 420 screams. Although this standardization of the scream to 800ms and 70 dB SPL might render some of the screams partly unnatural (i.e. alarming screams might have a higher natural loudness/intensity than non-alarm screams), this procedure ensured that all following analyses and perceptual experiments are not confounded by basic acoustic features of these screams due to vocalization characteristics and recoding conditions for single speakers. To avoid experimental confounds, we rated the exact standardization of the stimuli of higher importance for a straightforward interpretation of the stimuli compared to introducing a little bit less natural sounding screams. Furthermore, although these screams were largely acted rather than spontaneously expressed, acted screams seemed to be perceptually similar to natural screams [5,6], and thus provide a valid basis for affective communication research in human and nonhuman primates”.

This type of standardization is used in many previous experiments (e.g. [7,8]) based on the same rationale to avoid confounds. Scream vocalizations in general and across emotional types are already of loud and intense nature. We therefore compared the original intensity of our screams to the loudness of normalized screams:

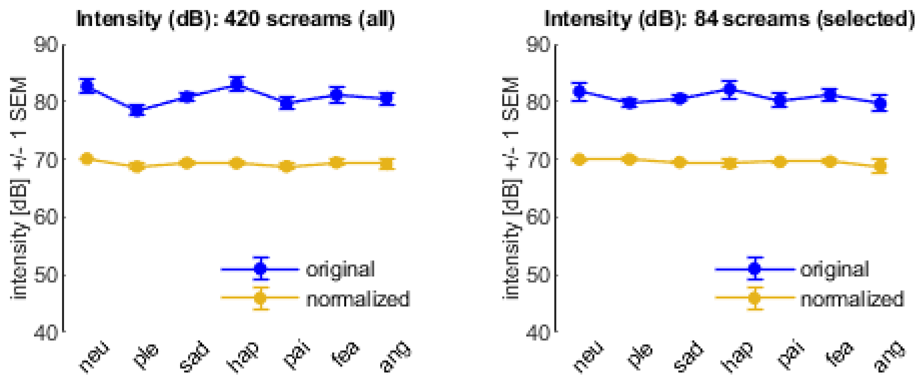

For the total 420 screams, we calculated the difference score between the original and the normalized scrams for each scream type, and applied a rmANOVA on these difference score to check if the normalization differentially affected the different screams types (i.e. if there was more loudness change in one scream type compared to the others). Using this analysis, there was a significant difference between the original and the normalized loudness across the scream types ($F_{6,354}$=13.664, p<0.001), Since all 420 screams were the basis of the alarm ratings presented in Fig. 2, we checked if there was a correlation between the loudness differences scores ([original - normalized]) and the alarm ratings for each scream. Performing a Pearson correlation between the loudness difference scores and the alarm rating did not reveal a significant relationship (r=-0.071, p=0.146), making it very unlikely that loudness is a dominant factor in scream perception, at least in our study.

P6: “Since all screams were normalized to a uniform loudness level of 70dB SPL, we furthermore tested of this loudness normalization influenced the alarm ratings of the screams. For each scream, we quantified the loudness level before and after normalization, calculated the loudness difference between the original and the normalized loudness, and performed a correlation analysis with the alarm ratings for each scream, which yielded a non-significant relationship (Pearson’s correlations, r=-0.071, p=0.146). This indicates that the alarm ratings were not influenced by the loudness normalization procedure”.

For the 84 screams, the difference score between loudness before and after normalization did not reveal a significant difference between scream types ($F_{6,66}$=1.375, p=0.238), which indicates the normalization to ~70dB was similar across all screams and is thus very unlikely to influence the behavioral and neural data in experiment 2-4.

P8: “Since also this selection of 84 screams were normalized to a loudness level of 70dB SPL, we tested if this normalization procedure affected the scream types differently. The loudness difference score between original and normalized screams did not differ across scream types 1w-ANOVA, 7 levels; $F_{6,66}$=1.375, p=0.238, η^2=0.09, and thus was unlikely to affect the behavioral data reported below”.

The authors now state on page 8 that there was no significant difference between non-alarm and alarm screams in accuracy, but on page 9, following the subtitle "Impaired perceptual discrimination of alarm compared to non-alarm screams" they claim to have found such a difference. I believe the authors refer to the F-Test that includes the "neutral" screams. However, put in this context and given the post hoc results, this sentence is simply wrong.

Response: In experiment 2, the accuracy data showed that alarm and non-alarm screams had lower recognition rates compared to “neutral” screams, with no differences between alarm and non-alarm screams. To better specify this finding, we now edited the first sentence in the section "Impaired perceptual discrimination of alarm compared to non-alarm screams" like this, p11:

“In experiment 2, the 6 generic scream calls as well as the three major categories of scream types (neutral, non-alarm, alarm) showed some selective differences in behavioral and sensitivity measures of their classification when all types of screams and choice options were available in a speeded multi-option decision task”.

One point I did not make sufficiently clear in my last review is that I believe that the observation of "two separate frequency bands" for affective screams simply results from the subtraction of MPS-values for the "neutral" screams. It is likely that the difference to "neutral" screams is rather a broader modulation for affective compared to neutral screams.

Response: Maybe we missed this point of the reviewer in the first review, but the reviewer mentions an important point here that the two ranges for significant modulation frequency bands result from the subtraction analysis with “neutral” screams This is obviously the case, and subtraction the MPS from other types of vocal stimuli might of course results in a different appearance of the difference MPSs. MPS for screams are typically compared against other types of vocalizations (e.g. [7]), in our case the most appropriate comparison condition was the “neutral” screams as the closest type of vocalizations. We highlighted this fact now in the manuscript, p6: “Generic screams seem to have a broader frequency range for temporal modulations, and the specific comparison to neutral screams especially highlighted these two frequency ranges”.

The authors did not reply to the question why loudness was included as a feature in the SVM when this parameter was actually standardized.

Response: We decided to include the mean intensity per scream into the SVM analysis because there was still some residual variance in loudness after normalization (i.e. minimal loudness difference between stimuli, because normalization to 70dB SPL is approximate), so this needs to be taken into account in the SVM analysis. Furthermore, even if the mean intensity is a rather flat features, it therefore does not

contribute much to the SVM estimation. Besides mean intensity, we of course took other intensity/loudness features into account, like intensity variation, because this is still an important feature for voice perception.

The error rate in the gender identification task has not been sufficiently explored. Although the category factor did not reach significance (p=.132) the highly significant effect of scream type was not followed up. It suggests an unexpected interaction between scream type and task performance in case scream type identification and gender recognition were really orthogonal tasks.

Response: We now also followed the significant effects concerning the accuracy rate for gender classification across all 7 scream tapes. We accordingly edited the manuscript, p12:

"During listening to these scream calls, participants performed a largely orthogonal gender decision task on the screams, with orthogonal meaning that the task is largely independent from the emotional quality of screams. The task was introduced to maintain the attention of the participants to the experiment, and is often referred to as implicit but still strong processing of the affective quality of the stimuli that leads to consistent brain activations [9,10]. Reactions times did not differ between all 7 scream types ($F_{6,174}$=2.371, p=0.094) nor between the 3 major categories of neutral, non-alarm, and alarm screams ($F_{2,58}$=0.769, p=0.414) (Fig. 5). The error rate was different between the 3 major categories of neutral, non-alarm, and alarm screams ($F_{2,58}$=3.747, p=0.041, but all posthoc comparisons with p>0.0531), but showed a difference between all 7 scream types ($F_{6,174}$=7.280, p<0.001, η^2=0.10), with gender classifications during pain screams showing higher accuracy compared to gender classification during anger, sad, happy, and neutral screams (all posthoc p's<0.030), and a lower gender classification accuracy during sad compared to fear and pleasure screams (all posthoc p's<0.018). Gender classification thus was equally performed in terms of speed and classification accuracy for the three major scream categories, but gender classification during the perception of certain scream types led to some specific better (pain) or worse performance (sad) compared to other screams. This could be partly based on the acoustic profile of these screams (i.e. pain scream acoustics might less obscure gender-related acoustic, while sad scream might obscure this information more), and partly on the emotional relevance of both scream types (i.e. for "physical" pain gender might be relevant to understanding the source of pain, while for "social pain" in sadness gender becomes a minor relevant feature) [11,12]".

On page 16 the authors overinterpret their findings as "specific neural pathways" and "specific decoding" without having tested specificity.

Response: The reviewer is correct that we did not test for specificity here. With the term "specific" we rather wanted to highlight that some form of scream processing takes certain neural pathways compared to other possible pathways. To avoid confusion here, we changed this sentence now, as found on p17:

"Thus, there seem to be certain neural pathways connecting subregions in the right auditory cortex for non-alarm scream processing as well as connecting the auditory cortex with the frontal cortex and the limbic system to decode positive meanings from screams".

The Figure Legends should include the number of observations (n=26, 33…).

Response: The number of observations is now included in the figure legends.

Reviewer #2:

I thank the authors for the careful revision and their responses to my comments. They raise some fair arguments and I agree with most. Yet, I still disagree with the strong claim that they are making that alarm screams have lower efficiency in the cognitive, neural, and communicative processing. It seems clear to me, that, based on the results presented by the authors, non-alarm screams have a higher discriminability advantage compared to alarm screams among themselves (among these three categories). This however,

does not mean that alarm screams have a disadvantage. As reviewer 4 and also the authors themselves point out, 'alarm scream categories have some primacy during misclassification of other scream types' , which seems contradictory with the claim that the authors are trying to make.

Response: We thank the reviewer for the positive feedback on our revised manuscript, and we appreciate the remaining comments and suggestions in this second review. We give our detailed response to the comments below, and we also tried to incorporate the suggested point at various locations throughout the manuscript.

We quickly wanted to include our response about the specific point that the reviewer mentions here in the first comment. Experiment 1 shows that all 7 scream types are acoustically very distinct, and there is no reason to believe that the acoustic quality of screams introduced a bias to not being able to accurately recognize one of the scream types. Based on the behavioral data that we obtained in experiment 2 and 3 in our study, we can say that alarm screams take the longest RT to be classified (Fig. 2a), they show the lowest perceptual sensitivity in the form of d’ (Fig. 1c), and it takes more RT (and humans make more errors) to discriminate within alarm than within non-alarm screams (Fig. 1e.). While it might be a topic of discussion if one can classify these behavioral patterns as “disadvantage” for alarm screams, they certainly do not speak towards an “advantage” in the perceptual classification of alarm screams.

Furthermore, the observation that alarm screams categories are often used during misclassification of all screams ('alarm scream categories have some primacy during misclassification of other scream types') can be a topic of debate. If alarm scream categories are often used during misclassification of any type of scream, this again does not speak for a processing “advantage”. A cognitive system usually wants to achieve high recognition accuracy, so introducing a factor in the recognition process that facilitates classification errors is surely not of “advantage” for a human recognition system. So, we are unsure what the reviewer means by stating that this ‘seems contradictory with the claim that the authors are trying to make’.

We of course can discuss if this “bias towards making classification errors” is of severe “disadvantage” for an organism, and we already discussed a potential positive benefit of these classification errors in the manuscript, p18:

“The only priority that alarming scream categories received was their frequent use during misclassifications of other screams in a multi-option classification task in experiment 2. This might resemble a natural threat perception bias that seems to be a cost-benefit efficient solution when balancing response options against potential sources of (non-)threat, especially under conditions of uncertainty [13]”.

To better highlight the positive effects we added an additional sentence to this section, p16:

“Although perceptual misclassifications seem to be of general disadvantage to any recognizing organism, these misclassifications as “alarm” screams might figure as a safer option in potential threat recognition”.

The authors argue in their response to one of my comment that 'discrimination within alarm screams can be lifesaving (e.g. mistaken an aggressive anger scream for a pain scream might cause you big harm, because you then tend to approach rather than to avoid the angry person/source).'; and 'organisms needs to decide quickly if a scream results from pain (help the other person; you do not need to run away from a person screaming out of pain) or from anger (run away from the angry person).'

This differential behavioural response to sounds of pain and anger (approach someone in pain and run away from an angry person) would likely not occur in most non-human animals, or even in humans exposed to a dangerous environment (except if the person in pain is a close relative, which was not the case in your experiment). In nature, a pain scream might indicate that there is a high danger around (e.g. predator) and that one needs to run away (not necessarily approach the person/animal in pain), at least if we consider how non-human animals might react. Therefore, I still believe that in a natural setting (and likely in our ancestors), the immediate, safest response to any alarming sound would be to run away, before 'higher' cognitive processes occur for a finer discrimination.

Response: The reviewer mentions an important point here, and we agree the proposed hypothesis (pain and anger screams make the perceiver to run away) has some appeal and we included a respective notion in the revised manuscript (see below). However, we also wanted to quickly mention that the idea that pain/anger elicit the same indiscriminate response in listeners seems a rather vague and evidence for this seems missing in the animal literature. If pain and anger screams would elicit the same kind of response (run away), it also then raises the question why both screams are acoustically so different.

First, we did a thorough paper search on the internet, and we did not come across any study about how animals behaviorally respond to the pain that they perceive in other conspecifics (approach or flight). There are recent studies about some animals that adapt their behavior to avoid pain in conspecifics (e.g. rats avoid pain to conspecifics during food choice [14]), and many animals show empathic responses to painful conspecifics. Also, if the predator is still present, animals/humans would rather scream in fear to alarm others to run away (suppress the pain scream), and only start painfully screaming if the predator vanishes. So there is scarce evidence in animal research that could substantiate the claim that animals run away from a conspecific screaming in pain. We would be honestly happy, if the reviewer could point us to the relevant literature, in case we missed an important line of research here.

Second, painful screaming in the presence of a predator seems only a very specific case. Humans painfully scream e.g. when they had an accident (with no predator involved), when having a stomach ache after eating bad food, or when having a neurological disorder causing pain. We think a normal human being would not run away from these situations when perceiving another person screaming from pain. Humans are quite empathic to the pain of others.

Third, our study was about scream perception in humans (and not in animals). Although there is of course a similarity how animals and humans respond to others' screams, there might also fundamental differences between the species (which we also indirectly show in this study, and this was also mentioned by reviewer #4). As humans, we therefore might not respond similarly as animals in certain contexts. At least, research in humans shows that humans rather approach a person in pain than running away from that person, and this might be a fundamental difference to animals, such that the animal literature is only partly useful here.

However, because the idea has some appeal, we now discuss it in the paper, e.g.:

P12: "In addition, more discrimination errors occurred for all negative screams (i.e. screams with a negative affective valence) when presented together with neutral screams (sad: t_{34}=3.358, p=0.005; pain: t_{34}=4.980, p<0.001; fear: t_{34}=3.435, p=0.005; anger: t_{34}=3.303, p=0.005) or with joyful screams (sad: t_{34}=7.615, p<0.001; pain: t_{34}=6.441, p<0.001; fear: t_{34}=7.213, p<0.001; anger: t_{34}=7.973, p<0.001); for angry screams when presented together with pleasure screams (t_{34}=2.652, p=0.025); and for neutral screams when presented together with joyful screams (t_{34}=3.787, p=0.002) (Fig. 4c-d)".

P19: "The behavioral data from the speeded classification tasks in experiments 2 and 3 thus seem to primarily point to a less efficient processing of alarm compared to non-alarm screams in these speeded classification contexts. This seems indicated by increased response times when classifying screams (Fig. 2a), a lower perceptual sensitivity when quantified with the d prime measure (Fig. 2c), and by higher response times and lower discrimination accuracy when discriminating among alarm screams (Fig. 2e). Also, alarm scream categories are more frequently chosen when humans misclassify screams (Fig. 2d). We already discussed the latter observation above of being of some advantage for an organism as this would be a safer choice option in case there is potential and suspected danger in the environment. Given the above description of the lower decisional efficiency in classifying alarm screams themselves, it might be that a critical function of alarm signals in general and of alarm screams in specific is to increase the alertness of the cognitive system to other ongoing events. Previous research has shown that alarm signals increase physiological responses in listeners [1,2], and that this facilitates the detection of separate targets [3,4]. This focus on secondary targets might distract the attention away from alarm screams, and might lead to increased response times for classifying the alarm scream itself and for discriminating it from other alarm screams".

The authors also argue that 'a stress response might explain increased RTs for alarm screams, but it does not explain higher error rates for alarm screams; if people take longer to classify alarm screams ("higher cognitive processing"), it does not explain why they make more errors (if people take longer to process sensory information, the usually get better in classifying the stimulus).' However, if I understand correctly, the higher errors (false alarms) were among alarm screams, showing that people find it hard to discriminate among these screams. Yet, they might still be able to quickly discriminate between alarm and non-alarm, which is what would be important for survival. Therefore, I think the following claim that the authors added should be revised: 'While this could explain the increased classification times for alarm screams (i.e. respond first, and then discriminate), it does not explain the higher error rates, which still point to a classification and discrimination disadvantage for alarm screams.'

Response: The reviewer is (partly) correct here in regard to some of our significant finings. Experiment 2 showed that humans do not make more errors when classifying alarm compared to non-alarm screams (Fig. 3a; see also p9: "… with lower accuracy for non-alarm ($p<0.001$) and alarm screams ($p<0.001$) than for neutral screams, but with no difference in accuracy for non-alarm and alarm screams ($p=0.192$) (Fig. 3a)"). If humans misclassify screams, they tend to choose the alarm scream categories (Fig. 3d). Experiment 3 showed that discriminating within alarm screams involves the longest RTs and the most errors (Fig. 3e-f). The latter results seem also the part the reviewer is referring to with this comment.

The reviewer makes a good point here that discriminating within alarm scrams might not be the highest priority in terms of survival issues as long as there is a "fast" and adaptive response to any alarm sound. The reviewer furthermore mentions that discriminating across alarm and non-alarm screams might be the most important issue for survival. This is an important point and this seems partly confirmed by our data, see Fig. 3e-f. Humans are faster and make fewer errors when discriminating non-alarm from alarm screams.

However, three other findings are at odds with the above notion. First, discriminating within non-alarm screams had the lowest RTs and the lowest error rates. So, if RTs and errors are an indicator for survival relevance, the discrimination between non-alarm screams is of higher importance according to our data than discriminating between non-alarm and alarm screams and than discriminating within alarm screams. Second, as shown in Fig. 4f-h, if humans had to directly discriminate non-alarm from alarm screams, it were again the alarms screams on which humans took longer to respond and made more misclassifications in some of the alarm with non-alarm scream combination (asterisk * in Fig. 4f-h, the red color indicates higher values for the [alarm – non-alarm] difference). For none of these combinations, we found that alarm screams were classified faster and with less errors. Third, in experiment 3 we even found that more discrimination errors occurred for all negative screams (i.e. screams with a negative affective valence, and thus including all alarm screams) when presented together with neutral screams. So it seems that even against neutral scream the alarm screams do not show a processing superiority. Altogether, even when humans had to directly discriminate an alarm from a non-alarm scream, the performance was still worse for the alarm screams.

To have a balanced view on the data obtained in experiment 3, and to include both the confirming and non-confirming findings in the revised manuscript, we revised the related section in the manuscript as follows, p12:

"Concerning the above-reported patterns, there might be the possibility that alarm screams do not need to be discriminated very quickly, because they only need to activate the perceiving organisms to indiscriminately respond to any potential threat. While this could explain the increased classification times for alarm screams (i.e. respond first, and then discriminate), it can only partly explain the higher error rates when humans classify and discriminate within alarm screams. An important notion might be that misclassifications with alarm screams often take the form of misclassifying them as another alarm scream, which could lead nonetheless to the same appropriate behavioral adaptions. Furthermore, instead of discriminating within alarm screams, the discrimination between non-alarm and alarm screams might be of higher relevance for survival. We indeed found that discriminating between non-alarm and alarm screams is overall faster and more accurate than discriminating within alarm screams (Fig. 4a-b). However, although these discriminations between non-alarm and alarm screams showed an overall better performance than

discrimination within alarm screams, it was still the alarm screams that revealed a worse performance compared to non-alarm screams in specific combinations between non-alarm and alarm screams (Fig. 4c-d). The latter observation still points to some form of classification and discrimination disadvantage for alarm screams".

I appreciate that the authors now also quantified the RTs to the alarm ratings presented in Fig. 1c, and found that people did not show faster RTs in the alarm rating for alarm compared to non-alarm or neutral screams. However, these new results also show that people were not slower for rating the alarm of alarm screams, suggesting no 'inferior processing efficiency' for these types of sounds.

Response: As requested by reviewer #1, we now also include the RT data from the alarm rating in the newly revised manuscript (see Fig. 2). We also have to note here, however, that the alarm rating experiment was not designed as a speeded classification experiment; for the ratings, participants were required to move a slide on a screen, and they could take their own time to do these ratings. This is why the RTs for the rating study are in the range of **~3.5s**, while for the speeded classification experiment is was in the range of **~1.5 s**. Finding no RTs differences in the rating experiment is thus an experimental result, but it did not originate from a speeded classification experiment. This however shows that RT data are task-dependent, in this is now clearly mentioned in the manuscript.

Overall, I still think that these results warrant publication, but I think the authors should be more cautious with how they interpret their results. For instance, I would tone down claims suggesting that alarm scream have some disadvantages, and develop potential alternative hypotheses further in the discussion. They could make clear, throughout the manuscript, that the impaired perceptual discrimination of alarm screams is among the three types of screams of this category (not between alarm and non-alarm screams).

Response: As requested by the reviewer we now broaden the discussion at many points in the manuscript to also highlight potential alternative hypotheses in the manuscript (see also our response to the comments above). On many occasions throughout the manuscript we also better highlight the task dependency of many of the findings, and we carefully toned down claims of a processing disadvantage for alarm screams when appropriate. However, many of our data still point to some form of processing disadvantage for alarm screams, and we think it valid, reasonable, and justified to clearly describe these findings also in the manuscript.

We also discuss the reviewers' notion "… that the impaired perceptual discrimination of alarm screams is among the three types of screams of this category (not between alarm and non-alarm screams)". As we outline above this notion is only partly correct, since we also have evidence in our study that alarm screams showed impaired performance when directly discriminated from non-alarm screams; see our response above.

In addition, it would be clearer if the term 'reaction time' could be changed to 'response time' or 'decision time', since this measure does not assess how fast people 'reacted' (they might have reacted before choosing an answer) but how fast people responded.

Response: We replaced "reaction time" with "response time" throughout the manuscript.

Reviewer #3:
[identifies himself as Harold Gouzoules]

I believe Frühholz et al. have improved the manuscript considerably and they have explicitly acknowledged virtually all of the concerns the reviewers outlined, myself included. In most instances, I think the authors have provided very thoughtful responses, but I will not weigh in here as to whether I think the issues raised by the other reviewers have been adequately addressed in this revision. Instead, I'll focus on the central issues I raised in my initial review that, I respectfully suggest, still need some additional attention.

Response: We thank Harold Gouzoules again for these very constructive comments on our revised manuscript, and thanks for the very positive feedback on our work and edits done in the first round of revision. We now have taken the remaining concerns very seriously and try to include more details about several of the experimental choices and designs.

My main concern remains with the source of the screams used in the experiment. My initial review noted the authors' description:
"We thus instructed the 12 participants to produce vocalizations of screams for each of the 6 types of generic screams, as well as to produce neutral screams on the basis of an intense vocalization of the vowel */a/*. For each type of scream, we provided short instructions to each participant to imagine several corresponding context(s) in which these screams are commonly produced (e.g. fearful scream: "You are being attacked by an armed stranger in a dark alley"; anger screams: "You try to intimidate an opponent"; joyful screams: "Your favorite team wins the World Cup"; pleasure screams: "You are screaming from sexual delight"). For each participant, we recorded 8 instances of each type of scream in an anechoic chamber with a Neumann TLM-102 microphone at a distance of ~1m from the speaker. From these 8 instances, we chose the 5 best recordings from perceptual judgment of recording quality …"

I think that the full set of prompts ("short instructions") given to the participants should be provided in the paper or supplemental materials. I assume those listed in the manuscript are just examples. What were the others for the different scream types? With a prompt such as "Your favorite team wins the World Cup" --- were the instructions to produce a scream the participant might imagine a rabid fan would make, or simply to produce a scream that they themselves would make? If the latter, what if the person is not a football fan, or even a sports fan? That would surely generate variation in the screams produced in this context that those involving, for example, the intense fear context might not show. Similarly, for the prompt "You are screaming from sexual delight," is the assumption that males and females are equally likely to scream in this context, or that, within one sex there are no differences in the tendency to scream during sex or in the nature of any screaming? How might the study's results be impacted by these issues?

Response: Thanks for this important comment. We used the "short instructions" to prime the participants for the vocalization recording with an example of a possible context for each type of scream.

We now provide more details about the recording context and the vocalization instruction, p23:

"For each type of scream, we provided two short instructions to each participant to imagine exemplary contexts in which these screams are commonly produced (e.g. fearful scream: "You are being attacked by an armed stranger in a dark alley"; anger screams: "You try to intimidate an opponent"; joyful screams: "Your favorite team wins the World Cup"; pleasure screams: "You are screaming from sexual delight"). These short instructions were provided such that participants would have two example situations, in which each scream type is typical (but not exclusively) expressed. This type of procedure with short instructions and examples is similar to previous research in the field [15]. Participants were instructed to imagine the emotional quality of some of the provided contexts, or similar contexts of the same emotional quality, and produce screams that would reflect their own vocal expression according to the emotional quality of this imagined context, but not necessarily of the specific person and/or event described in the short instruction. Before the actual recording, participants did a training session of about ~15min with the experimenter to ensure that the emotional context for each scream was understood correctly and that appropriate intensive vocalizations of a scream-like character (e.g. a harsh, loud, rough, and thrilling sound quality of the voice as described in the literature [7,16,17]) could be produced for each emotional context. Participants were instructed to deeply inhale before each vocalization to enable maximum glottal pressure for each single vocalization. Using these instructions and training, we recorded 8 instances of each type of scream for each speaker in an anechoic chamber with a Neumann TLM-102 microphone at a distance of ~1m from the speaker. From these 8 instances, we chose the 5 best recordings from the perceptual judgment of recording quality, vocalization length (800-900ms), continuous vocalization for the duration of the scream, and the perceptual impression of having a scream-like vocalization quality".

Furthermore, we have to note that for each type of scream, each speaker was asked to produce at least 8 different instances of vocalizations, and we chose the five best vocalizations based on the perpetual impression of scream-likeness on these recordings.

For the case of “sensual/sexual pleasure” the instructions were giving the example of “sexual delight”, as one prototypical example of sensual pleasure; the participants were allowed to also imagine other contexts that are related to sensual pleasure and that produce similar kind of screams. It was not assumed that male/female equally likely scream in this context, but regardless of the frequency both sexes do scream in this context, and we asked them to produce screams that they would produce in this context.

In my initial review I noted:
"The study's stimuli come from 12 volunteers asked to generate screams that they think would be associated with six different scenarios. Nothing is said about these participants (other their mean ages and that that they were healthy. Problematically, the authors assume that these 12 individuals have the acting talent to generate different screams as they were asked to do. …. Engelberg and Gouzoules note: "It is important to emphasise that the chance-level performances demonstrated here do not necessarily imply that acted and natural screams are identical in every respect, nor that screams from all actors are equally suitable for implementation in empirical research."
To this, Frühholz et al. respond:
"If screams are within the communication repertoire of every human, it should be that not only talented actors (NB: being an actor on TV might not necessarily imply that someone is talented) are able to produce valid vocalizations of screams, but every normal human being with precise instructions. We took care that scream vocalizations were produced at the intended level of screaming."
I have no idea what the authors mean when they say, "We took care that scream vocalizations were produced at the intended level of screaming." This is a vague statement. Could they please elaborate? The contention that "If screams are within the communication repertoire of every human, it should be that not only talented actors (NB: being an actor on TV might not necessarily imply that someone is talented) are able to produce valid vocalizations of screams, but every normal human being with precise instructions" is simply not convincing to me.
The authors' rib that not all TV actors are talented misses the point I was trying to make. We might indeed debate the talent of any individual TV actor, but there is no denying that these individuals are professionals under the guidance of a director and they have the opportunity more rehearsals and multiple takes. The authors are clearly reluctant to accept my point that it is an unfounded, and I suggest unreasonable, assumption that every human being is equally capable of producing different sorts of screams (or other nonverbal expression) that reflect natural usage, upon command …. no more than, despite having the general capacity for language, all humans can convincingly deliver a Shakespearean soliloquy. That screams are in the human vocal repertoire does not mean that they can be generated on command without training and experience …. that's the point and caveat we make (in Engelberg & Gouzoules 2018) about the potential to produce credible screams by professional actors.
Again, as I noted in my earlier review, the authors provide no details about how the screaming participants in this study were recruited? I think it's critical to the interpretation of the results to have more details. Did any have acting experience, for example? Are the results of the experiments comparable when examined as a function of the screams clusters contributed by the different screamers (i.e., were results associated with any of the scream contributors at odds with the general findings reported)?

Response: Producing emotional vocal expression on command indeed varies across individuals, and not all but many humans can vocalize screams to a certain degree on command (with screams probably being of less vocal complexity and needing less training than a Shakespearean soliloquy; our first vocalization when born is indeed a scream). In the description of the speaker sample we now include the information that all speakers were untrained speakers in a sense that none of them received formal acting training. P22:

“*Participants*. In experiment 1, 12 healthy volunteers (6 males; mean age 29.08 years, SD=5.66, age range 22-42) took part in the acoustic recording of 6 different types of screams, as well as a recording of neutral vocalizations as an intensive utterance of the vowel /a/ as a seventh category. All participants were healthy humans with no acting experience, training in acting, nor being professional actors”.

Furthermore, we included a limitation section in the revised manuscript including a point that highlighted the fact that in our experiment 1 only untrained speakers as a potential limiting factor in our study, and that these untrained speakers might not have produced screams at a full natural level, p21:

“Before summarizing our main findings across the experiments reported here, we want to discuss a few potential limitations in our study. First, the scream vocalizations in our study were produced and acted by a sample of normal healthy humans that were, unlike professional actors or trained vocal speakers, relative naïve to the production of screams on command. It might be therefore that the acted screams in our study were not of full natural quality as for screams triggered by natural cues [6]. However, based on previous descriptions about what makes a scream, our screams produced by naïve speakers seemed to be of similar scream-like voice quality [6,16,17]. Furthermore, the perceptual assessment of the scream recordings by a sample of independent listeners confirmed that most of the screams can validly convey the intended emotional meaning and that they were of an alarming quality to a variable degree”.

We however also have to highlight an additional point here. Although the scream screams were produced by untrained speakers, all recorded scrams that comprised the 420 screams underwent an evaluation produce as a perceptual assessment by another sample of human listeners. This evaluation procedure quantified how other humans perceived different types of emotional qualities in the screams. So this was an independent evaluation that screams were largely produced accruing to the intended emotional expression. Based on these perceptual evaluations, the 84 selected were chosen that received a relatively coherent emotional classification across the raters at a high level of recognition accuracy. Given this evaluation, we not only trusted the speakers to have produced the intended emotions in screams, but a sample of n=26 participants gave their perpetual impression, based on which we produced with the selection of stimuli.

In the response to this issue the authors go on to say:
"Furthermore, give the acoustic similarities our screams to screams reported in other papers, we are confident to have a recorded a scream database with our speakers that is of a valid nature."
This is also vague. To my knowledge, other papers have not explored in detail screams associated with the variety of contexts examined in the Frühholz et al. study, so how can the authors make this claim? Please elaborate with specific details.

Response: To ensure that our scream recordings were similar in their scream “likeliness” we compared our scream recordings with what has been described on the acoustic and perceptual properties of screams in the literature [7,16,17]. Furthermore, the web is full of videos and demonstrations of acted and (presumably) natural screams, also of high quality acted screams that the reviewer refers to. So, we first confirmed the scream-likeliness by comparing the acoustic appurtenance of our scream with descriptions in the literature, p24:

“Before the actual recording, participants did a training session of about ~15min with the experimenter to ensure that the emotional context for each scream was understood correctly and that appropriate intensive vocalizations of a scream-like character (e.g. a harsh, loud, rough, and thrilling sound quality of the voice as described in the literature [7,16,17]) could be produced for each emotional context”.

Furthermore, amongst other acoustic features, we also quantified a complex acoustic feature that is referred to as “vocal roughness” [7] and that seems specific (although not exclusive) to human screams. Quantifying this roughness features with MPSs indicated that also our scream recordings had this roughness feature (Fig. 1b).

Finally, we performed a perceptual assessment experiment with n=26 listeners to rate the emotional quality expressed in these screams. This assessment confirmed that the intended emotional quality can be perceived with above chance level in these scream recordings (Fig. S1). Our SVM analysis also confirmed that the different scream types can be acoustically separated to a high degree (Fig. 1d-e)

Based on these data, we are confident that the scream recordings are of valid nature, concerning both the general scream quality of the encodings, but also the differential emotional quality of the screams.

The authors continue their response with:
"Within the field of voice signaling research there is a big discussion if studies should use more natural vocalizations (with the disadvantage of having sampling biases, noisy backgrounds, unequal quality between signal categories, etc.) or using acted and recorded voice signals in the lab. Only the latter allows a more precise sampling of vocalizations and database creations, precise vocalization instructions, clean recordings, and equal sampling of vocalizations across categories. Since in this study we were interested in conducting precise psychoacoustic experiments and also precise neuroimaging experiments (which can be easily affect by degraded stimulus quality), we opted for using acted scream vocalizations, given that there are perceptually very close to natural screams (see quote above)."
I am, of course, aware of this issue in the literature (we review it briefly in Engelberg & Gouzoules 2018), and I appreciate that it has major implications for study design, and also for much significant theory that underpins ideas about human communication. However, the choice to use acted renditions of screams generated by (presumably) untrained participants is nonetheless an issue that should not remain unaddressed in the manuscript. I would ask that the authors, at the very least, acknowledge the possibility that an inability (by all, or some participants, or males, or female …) to produce naturalistic screams for the contexts described in the prompts might have impacted the results. To some degree, this question can be explored in the data, and I encourage the authors to examine this in whatever ways possible.

Response: As mentioned above, we now included a limitation section in the manuscript that also discusses the limitation in our study that screams by naïve speakers were not produced to a full natural level, p18:

“Before summarizing our main findings across the experiments reported here, we want to discuss a few potential limitations in our study. First, the scream vocalizations in our study were produced and acted by a sample of normal healthy humans that were, unlike professional actors or trained vocal speakers, relative naïve to the production of screams on command. It might be therefore, that the acted screams in our study where not of full natural quality as for screams triggered by natural cues, but the perceptual assessment of the scream recordings by a sample of independent listeners confirmed that most of the screams can validly convey the intended emotional meaning and being of alarming quality to a variable degree”.

Direct comparison to natural screams is difficult, as this would require the same type and number of screams produced by the 12 speakers in natural contexts. Of course, we could compare our data to other scream databases given that they would include the same range of scream types as used here. A comparison to other databases always has the downside that we would need to compare acoustic profiles across different speakers, which is not a recommended procedure for voice acoustics.

I have just a few other comments, in particular, about the summary of the comparative literature that the authors have now included in the manuscript. They say:
"Screams by lower ranking animals help to recruit support from allies [14,15], while higher ranking animals scream to intimidate the lower ranking [16]."
This is actually more complicated. Screams are not always used by dominants in agonistic encounters: there are separate and distinct threat vocalizations (at least in the various macaque and baboon species) that are acoustically very different from screams. But when challenged by a lower-ranking opponent, high-ranking individuals will revert to specific screams (we designated them as "arched screams") that are used to recruit support from matrilineal family members.

Response: thank you for this specification. We now edited this sentence to read like this, p3:

“Screams by lower-ranking animals help to recruit support from allies [18,19], while higher-ranking animals scream to intimidate the lower-ranking animal when challenged by this lower-ranking opponent [20]”.

Finally, in response to the additional literature suggestions I made, the authors note that " …. this list seems limited towards the reviewers' own work." I apologize if my literature suggestions came across as trying to promote my research. I think it's fair to say that ours is the primary extended body of work on animal

screams. A key takeaway is that that "alarm" is not the only thing communicated by nonhuman primate screams (see above observations about the use of screams by domain monkeys). Even in nonhuman primates, screams are more complicated acoustically, and in their contextual usage, than in most other taxa (acknowledging, of course, the authors' that the broad context of monkey and ape screams, unlike for humans, is agonistic in nature … a point I have been making in print and publicly, in interviews available on the web, for years).
Harold Gouzoules

Response: Agreed. The papers published in the Gouzoules group are of course a valuable basis for our experiments and also for the current paper.

Reviewer #4:

Fruhholz et al present a comprehensive investigation on screams. In particular, they extend the investigation to non-alarming screams and present a series of behavioral and neuroimaging studies. They observe that alarming scream are processed less efficiently and lead to smaller BOLD responses. Overall this is an impressive study, nicely written and very comprehensive. The results run contrary to expectations, which make the study the more interesting and potentially impactful. Because results are somehow unexpected, caution on the interpretation and duly ruling out alternatives is particularly important. Along those lines, I would like to understand better the fMRI study and in particular the effect of the null event (baseline) as well as neural adaptation. Below I provide a further description of those issues and other that the authors might consider.

Response: we thank the reviewer for this largely positive evaluation of our manuscript, the arbitrating comments, and the valuable ideas on how to improve our manuscript. In the following, we give our detailed responses to the comments.

I was very surprised to see in the fMRI results such a strong activation for the neutral stimuli. Can the authors comment in this surprising result and on how that speaks for the efficiency of the fMRI design.

Response: The reviewer mentions an important point here that needs to be carefully addressed. However, we have to mention that "neutral" stimuli did not reveal the overall strongest response, but only in comparison to some other scream types. Specifically, neutral screams did not reveal stronger activity when compared to pleasure, sad, and joy screams, but only when compared to pain, fear, and anger screams.

So activity for "neutral" screams was somewhere in the middle between the other generic scream types, and did not reveal the strongest activation as implied by the reviewer comment. Furthermore, "neutral" screams were only "neutral" when compared to the other generic scream, but neutral screams were still intensive vocalizations. Given that neural activity for neutral screams was differentially located within the other types of screams is not an uncommon observation and does not therefore invalidate the experimental design. As mentioned throughout the manuscript, we carefully selected the scream stimuli for the fMRI experiment, matched them in terms of loudness, and the trial order was randomized across blocks and participants. Furthermore, there is no *a priori* reason to believe that neutral trials cannot elicit higher activity than emotional trials; this also seems to depend on the neural region investigated. So, observing higher activity for neutral trials does not *per se* invalidate the efficiency of a design. It can be a valid experimental observation.

We discuss this point also in the response to the next comment of reviewer #4.

One alternative for the low responses observed for alarming sounds is neuronal adaptation. A known physiological fact is that stronger initial responses lead to smaller subsequent responses. Since neural adaptation can explain in part the surprising low responses reported in the fMRI, I invite the authors to

provide an analysis taking into account the serial order of the conditions. Here, one can compare the amplitude of the response for each category for the first, second, third, etc presentation.

Response: We have to note that “low responses” for alarming sounds were not found in general, but only in comparison to other types of screams, especially in comparison to non-alarm screams and neutral screams (Fig. S3). The reviewer argues that this might be caused by neural adaption, presumably by neural suppression effects. Since “low responses” were found differentially across the scream types, these neural adaption effects then also need to be selective for certain scream types, especially for the alarm screams.

To assess of neural adaption effects caused the lower activity for alarm compared to neutral screams, we divided the trials for each scream type and block into two halves, and compared the activity in both halves for the generic screams against neutral screams (e.g. first half [anger – neutral, second half [anger - neutral]). If neural adaption would occur selectively for alarm screams the negative difference to neutral screams should be higher in the second half than in the first half. For this analysis, we extracted the signal in the various regions (i.e. region of interest analysis) that we found active during the different contrasts performed. This resulted in the plot below:

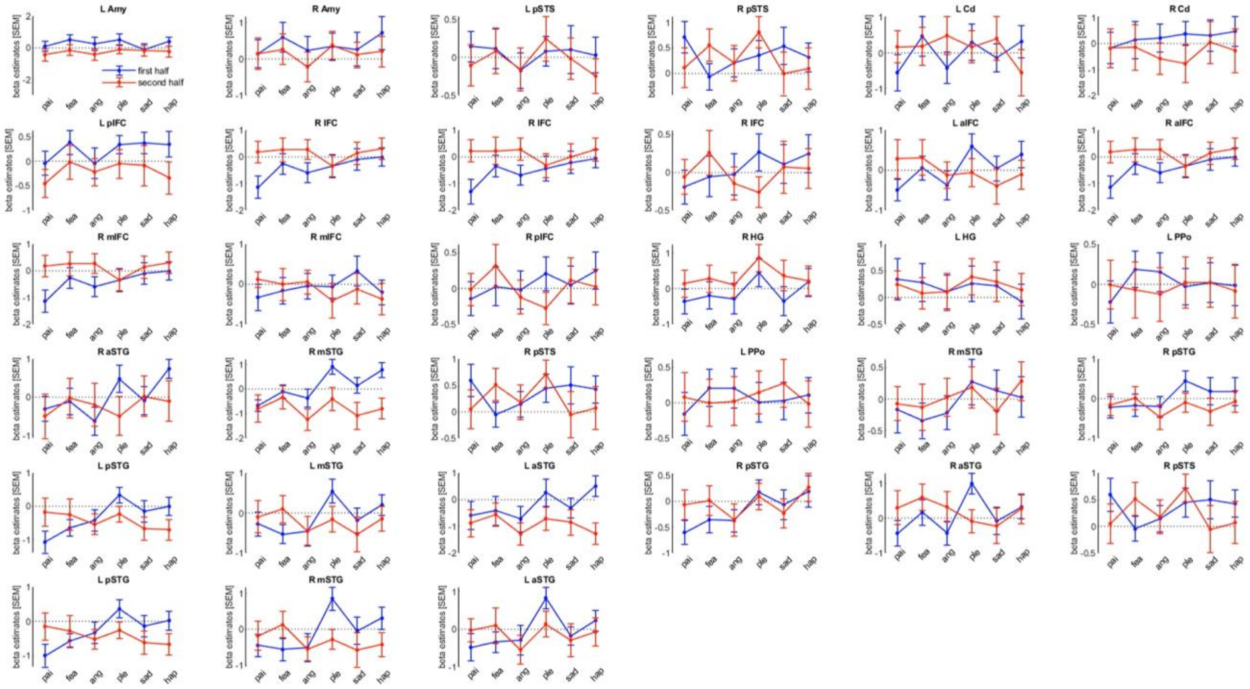

It seems like none of these data point to an apparent neural adaption effect especially for alarm sounds; in this case, the red line (second half) for pain, fear, and anger should be much below the blue line (first half).

The same analysis was performed by splitting the first appearance of a single scream from the second appearance of this scream. Again, the data do not indicate any neural adaptation effects that are specific to alarm screams.

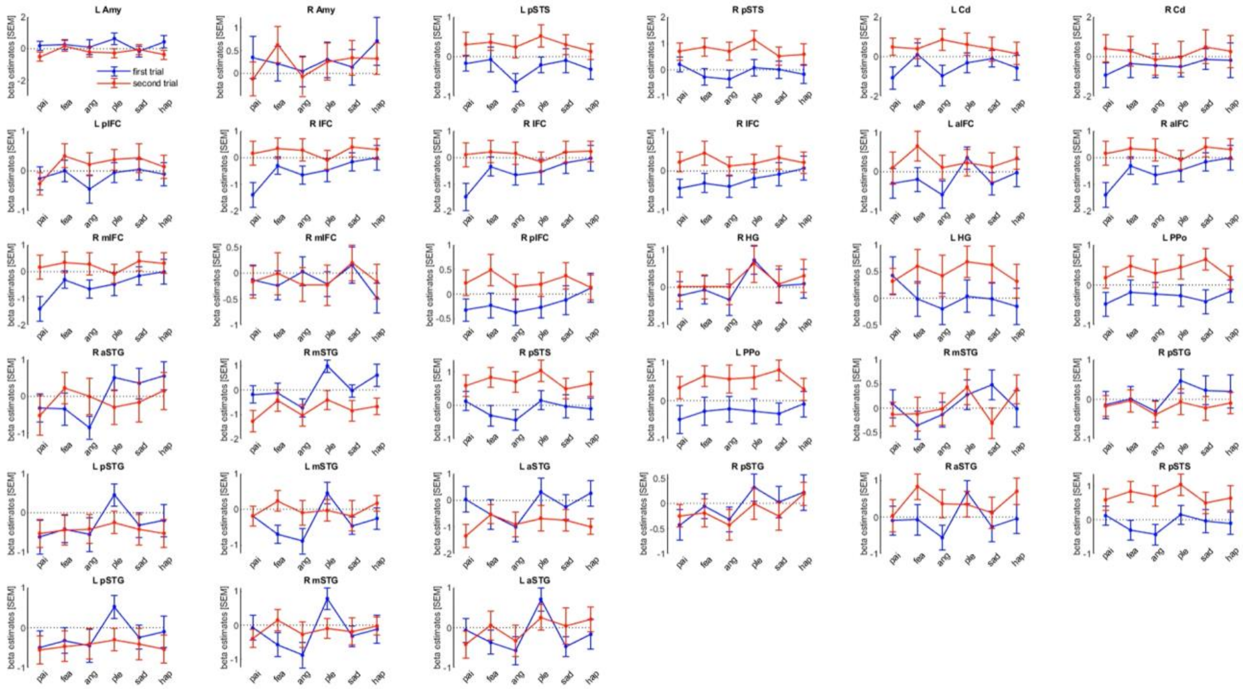

Therefore, it is highly likely that neural adaptation effects were causing lower activity for alarm sounds observed in our study.

Experiment 2 and 3. Please provide the intrasubject consistency (reliability when responding twice to the same stimuli), as well as intersubject agreement per stimuli and categories.

Response: The reviewer mentions an important point here. However, instead of including measures of ICC in the manuscript, we now included data from a full replication of experiment 2 in the manuscript, see Fig, 3. We hope that these data show that our results are highly replicable using different sets of selected screams. Also including ICC measures here would overload the already extended and fully packed data analysis with additional statistical values.

Experiment 4: Provide statistic for the reliability of the results comparing the post experimental test in experiment 4 with those obtained in the other studies. How replicable are the behavioral results across the two populations of subjects?

Response: The data from the post-experimental rating in experiment 4 is shown in Fig. S1c. This post-experimental rating was done by participants (n=30) that took part in the fMRI part of experiment 4. These participants performed the rating on the 84 selected stimuli taken from the total of 420 stimuli, and the rating was done in a non-speeded manner. During the evaluation of the total of 420 screams, another independent sample of n=26 participants rated all scream along the same dimensions (Fig. S1a). Related to this rating by the n=26 participants, we also plotted the ratings results of those 84 stimuli, that were selected for experiments 2-4.

So the only possibility to compared data shown in Fig. S1c would be a comparison for data shown in Fig. S1a or Fig. S1b. The data plotted in Fig. S1a-c show that the pattern of results is very similar across the three plots, including some minor differential effects. In the description to Fig. S1 we now report general statistical analyses that we similarly also applied to other behavioral data reported in the manuscript.

Methods:
Experiment 1: describe the subject recruited: where those naïve subject, or professional actors?

Response: The participants vocalizing the screams were normal humans with no acting experience, and none of them was a professional actor. This information is now included in the manuscript in the section where we describe the participant sample. All participants were given explicit instruction on how to vocalize the screams. A more detailed description of the vocalization instructions is now given in the manuscript, p22:

"In experiment 1, 12 healthy volunteers (6 males; mean age 29.08 years, SD=5.66, age range 22-42) took part in the acoustic recording of 6 different types of screams, as well as a recording of neutral vocalizations as an intensive utterance of the vowel /a/ as a seventh category. All participants were healthy humans with no acting experience, training in acting, nor being professional actors".

As there is no apriori reason to think that the authors have exhaustively cover all possible screams, I suggest to modify the following sentence " to comprehensively cover all possible screams…" to some like "to cover a broader range of possible screams".

Response: This sentence was changed accordingly.

Page 24. Using how quality headphones modify for using high quality…

Response: This was changed accordingly.

Provide descriptive statistics for missing trials, where those equally distributed across all the studied categories?

Response: The number of missing trials was very low with 6.53% (experiment 2), 1.55% (experiment 3), and 1.87% (experiment 4) of missing trials. These numbers is at a floor level and of minor proportion, ranging sometimes to zero percent. The relative flooring of the percentage of missing trials usually does usually precludes statistical tests, but instead, we now report the range of the percentage of missing trials across scream types of task conditions in the manuscript.

RT - outlier removal. What there any procedure to remove outliers?

Response: We did not include a formal outlier analysis for behavioral data. First, for the evaluation experiments, participants were provided with "infinite" time as they wanted for answering the different rating scales. The evaluation experiments (Fig. 1c, Fig. S1) were completely self-paced, which also resulted in a large variation of response times. For the speeded classification experiments, we defined a maximum response time window that was controlled by the experimental script. The maximum response time window ensured that participants responded in a given time frame. If no response was recorded in this time frame, the trial was classified as a missed trial. Given this experimentally controlled response time window, we ensured that responses were made in a defined time interval. This procedure usually ensures that extreme outlier RTs are not present in the data. Also, if you give participants a certain time window to respond, you should allow all response times in this window as valid trials.

For the calculation of d' explain what was taken as the noise and the signal distribution for each contrast.

Response: For calculation d rime measure, noise and signal distribution refer to the general notion of the d prime measure, for behavioral data, the d prime can be calculated according to:

$d' = Z(\text{hit rate}) - Z(\text{false alarm rate})$

We took this formula, which is common in sensitivity calculation with behavioral classification data, as the basis for calculating the d prime. Based on the formula above we used this Matlab function to calculate the d prime measure based on our observed correct classification rate for each scream type in relation to the false alarm rate for each scream type:

https://ch.mathworks.com/matlabcentral/fileexchange/47711-dprime_simple-m

Hit rates and false alarm rates are reported in many of the figures in our manuscript. This is now more clearly mentioned in the manuscript, p24:

“From the correct classification for a scream category and the level of false alarm rates that used a certain scream category, we calculated the d′ measure as an indicator of the sensitivity and detectability of a certain scream type. The d’ measure was calculated according to the formula $d' = Z(\text{hit rate}) - Z(\text{false alarm rate})$. The d′ measures were subjected to the same ANOVA analyses as described above*.

Specify what is the implicit baseline for the fMRI study. For the methods, it wasn't clear whether a null event was incorporated in the fMRI design.

Response: Null events are commonly included in auditory experiments that use a sparse sampling protocol, for fMRI experiments. In continuous fMRI sampling protocols, all scans that are not modeled in the first-level GLM are subjected to the calculation of the baseline. This is the default setting as implemented in the SPM12 software that we used for the fMRI data analysis. So, the implicit baseline in our case was calculated on all scans that were not modeled with events in the GLM design. For continuous sampling protocols, null events are not necessary, since non-modeled scans are the “null events”

provide length of each blocks

Response: We guess that this comment refers to the length of the blocks during the fMRI experiment. For the voice localizer scan we acquired 470 scans per participant, and for each block of the main experiment we acquired 565 scans. This information was now added to the manuscript. The length of the block follows the formula “nb scan * TR”, resulting in a duration of the voice localizer scan of 12.53min and for each block of the main experiment of 15.06min.

Figures: Fig 2. B and C labels are mixed up

Response: This is true, and was now corrected.

Results: F statistics: some of the reports of degrees of freedom do not check with the reported sample size. Please revise.

Response: We carefully checked all df’s for the F-tests and corrected all minor issues in the revised manuscript.

ARBITRATOR COMMENTS FROM REVIEWER #4 [lightly edited]:

Now I had the chance to review the paper. It is indeed a very comprehensive one and it is clear the authors have put a lot of work into it i.e., constructing new stimulus materials, behavioral studies and also fMRI.

however after reading the manuscript, the reviewers comments and the replies I must admit that I am on the reviewers' side.

Overall, I believe the points all 3 reviewers are making are reasonable and fair. They do raise serious concerns that muddle the interpretation of the data; and in my view the authors could minimally have toned down the interpretation and/or best run a control study.

Response: We thank reviewer #4 and arbitrator for these honest, constructive, and motivating feedback on our manuscript. In this second revision, we thoroughly revised our manuscript according to all reviewers’ and arbitrator comments. We especially now also include a replication experiment of our original experiment 2 as a kind of control study to show that our data are largely robust against any selection of stimuli.

P27: “We replicated experiment 2 with another independent sample of 29 healthy volunteers (18 males; mean age 25.96 years, SD=4.30, age range 18-32), but using a different selection of scream stimuli”.

P28: “As mentioned above, we repeated the same experiment with another sample of participants, but using a different selection of 84 stimuli out of the total 420 stimuli. This replication was done to show that the selection of a certain subsample of screams does not affect the general pattern of results obtained in experiment 2. Out of the 420 screams we thus selected another sample of 84 screams that overlapped with the original selection by 25%. This overlap was due to the fact that the selection procedure was guided by the constraint that the selected screams should not have a difference in their base recognition rate. Out of the total number of n=9.1626e+89 possible solutions for selecting 84 stimuli out of 420 screams, we ran n=10'000'000 simulations, and it was not possible to obtain a new selection of n=84 stimuli that did not include some of the previously selected stimuli, including the restriction that the base recognition rate is not significantly different between the seven stimulus categories. The minimal solution was to allow an overlap of selected screams of ~25% between the first and the second selection. The second selection of 84 screams again did not show significant differences in the recognition rate across scream types ($F_{6,150}$=1.596, p=0.182). Mean arousal level differed across all 7 scream types ($F_{6,150}$=45.137, p<0.001), across the 6 generic screams ($F_{5,125}$=10.551, p<0.001), and between neutral, alarm, and non-alarm screams ($F_{2,50}$=72.327, p<0.001)”.

We have to note here that the original submission was reviewed by 4 and not by 3 reviewers, as mentioned here by the reviewer. We just wanted to mention this here, because we guess that reviewer #4 in the second round of revision is not the same reviewer #4 from the first round of revision. So we just took the comments of the new reviewer #4 as new comments from a new reviewer.

Moreover, the paper is quite vague, or silent on critical aspects of the studies, i.e., the methods. For replicability, specially of this new and unexpected findings, the authors should make an effort to better and more extensively describe the experimental procedure.

Response: We now included much more details about the methodological aspects of our studies in the revised manuscript. We especially now include more details about the scream recoding procedure (experiment 1) and also the details of the fMRI experiment (experiment 4).

P23: “*Experimental design*. We invited participants to vocalize 6 different types of screams. These types of screams were chosen to cover a broad range of possible screams that humans vocalize in certain emotional states. A previous report on the acoustic and neural processing of screams identified screams as being largely only of a negative fearful nature [5] to signal alarm to other conspecifics [21] on the basis of a certain and unique acoustic feature of “roughness” (i.e. high-frequency spectro-temporal acoustic modulations). Although fearful screams are a prominent example of human screaming, humans produce vocal screams of a rough acoustic nature not only in the emotional state of fear, but also in a variety of emotional states referred to as “pleasure,” “sadness,” “joy,” “pain,” “fear,” and “anger” states. These different types of screams can be classified as either positive (pleasure, joy) or negative screams (sadness, pain, fear, anger), which we refer to as the factor “valence.” Furthermore, screams can be classified as either alarming vocal signals (pain, fear, anger) or as non-alarming vocal signals (pleasure, sadness, joy), which we refer to as the factor “alarming quality.” Thus, screams are not only limited to negative and alarming screams of fear, but they can also be positive and of a non-alarming nature.

We thus instructed the 12 participants to produce vocalizations of screams for each of the 6 types of generic screams, as well as to produce neutral screams on the basis of an intense vocalization of the vowel /a/. For each type of scream, we provided two short instructions to each participant to imagine exemplary contexts in which these screams are commonly produced (e.g. fearful scream: “You are being attacked by an armed stranger in a dark alley”; anger screams: “You try to intimidate an opponent”; joyful screams: “Your favorite team wins the World Cup”; pleasure screams: “You are screaming from sexual delight”). These short instructions were provided such that participants would have two example situations, in which each scream type is typical (but not exclusively) expressed. This type of procedure with short instructions and examples is similar to previous research in the field [15]. Participants were instructed to imagine the emotional quality of some of the provided contexts, or similar contexts of the same emotional

quality, and produce screams that would reflect their own vocal expression according to the emotional quality of this imagined context, but not necessarily of the specific person and/or event described in the short instruction. Before the actual recording, participants did a training session of about ~15min with the experimenter to ensure that the emotional context for each scream was understood correctly and that appropriate intensive vocalizations of a scream-like character (e.g. a harsh, loud, rough, and thrilling sound quality of the voice as described in the literature [7,16,17]) could be produced for each emotional context. Participants were instructed to deeply inhale before each vocalization to enable maximum glottal pressure for each single vocalization. Using these instructions and training, we recorded 8 instances of each type of scream for each speaker in an anechoic chamber with a Neumann TLM-102 microphone at a distance of ~1m from the speaker. From these 8 instances, we chose the 5 best recordings from the perceptual judgment of recording quality, vocalization length (800-900ms), continuous vocalization for the duration of the scream, and the perceptual impression of having a scream-like vocalization quality".

The reviewers have raised some of these points, but the reply was not satisfactory. For instance,
1) stimulus materials: what are the specific instructions given to the participant to prompt the stimuli? Who where those subjects: professional actors or naive participants.

Response: Please see our response above; see also p22:

"*Participants*. In experiment 1, 12 healthy volunteers (6 males; mean age 29.08 years, SD=5.66, age range 22-42) took part in the acoustic recording of 6 different types of screams, as well as a recording of neutral vocalizations as an intensive utterance of the vowel /a/ as a seventh category. All participants were healthy humans with no acting experience, training in acting, nor being professional actors".

P21: "Before summarizing our main findings across the experiments reported here, we want to discuss a few potential limitations in our study. First, the scream vocalizations in our study were produced and acted by a sample of normal healthy humans that were, unlike professional actors or trained vocal speakers, relative naïve to the production of screams on command. It might be therefore that the acted screams in our study were not of full natural quality as for screams triggered by natural cues [6]. However, based on previous descriptions about what makes a scream, our screams produced by naïve speakers seemed to be of similar scream-like voice quality [6,16,17]. Furthermore, the perceptual assessment of the scream recordings by a sample of independent listeners confirmed that most of the screams can validly convey the intended emotional meaning and that they were of an alarming quality to a variable degree".

2) fMRI design: I couldn't tell whether they have a null event or not, and/or what was the baseline. It is rather surprising that the highest activations are for the neutral stimuli, this makes me to suspect strong neural adaptation. If so, then an important part of the result might just be confounded by the design

Response: The reviewer mentions an important point here, but we have to note that we did not find the highest activation for the neutral trials, but for the trials including non-alarm screams, see Fig. S2. Concerning the issue of neural adaption, please see our response below.

3) standarization: Reviewer 1 makes a very good point that the standardisation could explain part of the results. The authors have decided to argue but this is a serious point as all results could boil down to alarm scream being higher in loudness and the brain normalising for this. If this feature is removed from the stimuli then they loose the critical property. The authors have remove that for good reasons, as it may confound the fMRI result. However, a better alternative could have been to include it in the GLM and investigate it explicitly.

Response: Stimuli normalization is a very critical issue in psychoacoustic studies, and many psychoacoustic and fMRI studies do normalize their stimuli In order to avoid confounding effects. As we have now specified in the response to reviewer # 1, loudness was a very unlikely feature that influenced the data.

P6: "Since all screams were normalized to a uniform loudness level of 70dB SPL, we furthermore tested of this loudness normalization influenced the alarm ratings of the screams. For each scream, we quantified the loudness level before and after normalization, calculated the loudness difference between the original

and the normalized loudness, and performed a correlation analysis with the alarm ratings for each scream, which yielded a non-significant relationship (Pearson's correlations, r=-0.071, p=0.146). This indicates that the alarm ratings were not influenced by the loudness normalization procedure".

P8: "Since also this selection of 84 screams were normalized to a loudness level of 70dB SPL, we tested if this normalization procedure affected the scream types differently. The loudness difference score between original and normalized screams did not differ across scram types 1w-ANOVA, 7 levels; $F_{6,66}$=1.375, p=0.238, η^2=0.09, and thus was unlikely to affect the behavioral data reported here".

4) stimuli subselection: from the 420 stimuli that were initially created a 1/4 is used for the further studies but the rationale for selecting those is poorly described. Furthermore, given the surprising results, demonstrating that the behavioral results are not driven by the subselection would have been a much better choice.

Response: The rationale for selecting the 84 screams out of the total 420 screams is now described with all details in the manuscript. Specifically, we choose screams with a similar base recognition rate from the evaluation study, such as to not bias any accuracy results in the speeded classification task in experiments 2-3.

P27: "*Experimental design*. From the acoustic scream recordings of experiment 1, we selected 84 screams (3 male, 3 female speakers) with 2 instances of screams per category. Stimuli were selected from the results of the perceptual assessment of screams in experiment 1, such that no significant differences in the recognition rate across scream types ($F_{6.150}$=1.895, p=0.117) were found for this selection. Mean arousal level differed across all 7 scream types ($F_{6,150}$=51.065, p<0.001), across the 6 generic screams ($F_{5,125}$=11.647, p<0.001), and between neutral, alarm, and non-alarm screams ($F_{2,50}$=84.558, p<0.001)".

P28: "As mentioned above, we repeated the same experiment with another sample of participants, but using a different selection of 84 stimuli out of the total 420 stimuli. This replication was done to show that the selection of a certain subsample of screams does not affect the general pattern of results obtained in experiment 2. Out of the 420 screams we thus selected another sample of 84 screams that overlapped with the original selection by 25%. This overlap was due to the fact that the selection procedure was guided by the constraint that the selected screams should not have a difference in their base recognition rate. Out of the total number of n=9.1626e+89 possible solutions for selecting 84 stimuli out of 420 screams, we ran n=10'000'000 simulations, and it was not possible to obtain a new selection of n=84 stimuli that did not include some of the previously selected stimuli, including the restriction that the base recognition rate is not significantly different between the seven stimulus categories. The minimal solution was to allow an overlap of selected screams of ~25% between the first and the second selection. The second selection of 84 screams again did not show significant differences in the recognition rate across scream types ($F_{6,150}$=1.596, p=0.182). Mean arousal level differed across all 7 scream types ($F_{6,150}$=45.137, p<0.001), across the 6 generic screams ($F_{5,125}$=10.551, p<0.001), and between neutral, alarm, and non-alarm screams ($F_{2,50}$=72.327, p<0.001)".

[in the reviewer comments], I am providing you with further aspects for the authors to consider beyond the ones that have been already raised.

Response: We did our best to address all the points raised by reviewer #4 in the list of comments.

Overall, this is a nice manuscript; and the results (if true), are interesting. If the authors could provide a more compelling revision (addressing the concerns raised by the reviewers for instance by explicitly acknowledging the effect of the task), it could be nice paper for PloS Biology.

Response: Thank you again for this positive feedback on our manuscript. All task effects are now explicitly described and discussed in the revised manuscript.

P6: "In an additional analysis, we tested for response time difference for the alarm ratings (Fig. 2b) and found no difference across all 7 types of screams ($F_{6,132}$=2.192, p=0.098, η^2=0.01) nor between the three

major categories ($F_{2,44}$=3.015, p=0.084, η^2=0.01). Thus, these self-paced alarm ratings were performed with a similar response latency across scream types, but with overall relatively slow responses (>3s) compared to the speeded classification tasks as we report below".
P8: "Having shown in experiment 1 that human screams are not limited to alarming screams signaling threat, but rather show an acoustic diversity of at least 6 different types of screams, we investigated in experiment 2 how accurately human listeners can perceptually discriminate and classify these different scream types in a speeded classification task".

P17: "Only in the speeded classification task, but not in the self-paced rating task that included making ticks on a visual analog scale on the screen, we found indication for some lower processing efficiency of alarm compared non-alarm screams. This indicates that this lower processing efficiency for alarm screams only occurs when humans have to make fast judgments on the scream signals perceived, and these fast judgments seem critical in some contexts".

P18: "This additionally suggests that alarm screams surprisingly need more processing effort during simple scream discrimination with only two choice options in a speeded discrimination task, and that rather non-alarm and positive screams are more efficiently processed during this perceptual discrimination of two types of screams".

P21: "Second, we found indications of some lower processing efficiency for alarm compared to non-alarm screams especially in speeded classification tasks including complex 7-alternative-forced choice options tasks as well as in a simpler two-option discrimination task. No such differences were found in a self-paced and non-speeded rating task that asked humans to rate screams along their alarm level. The lower processing efficiency for alarm screams thus seem task-dependent and seems to be observed in contexts that require speeded decisions".

REFERENCES

1. Bach DR, Schächinger H, Neuhoff JG, Esposito F, Salle F Di, Lehmann C, et al. Rising sound intensity: An intrinsic warning cue activating the amygdala. Cereb Cortex. 2008;18: 145–150. doi:10.1093/cercor/bhm040
2. Bach DR, Buxtorf K, Grandjean D, Strik WK. The influence of emotion clarity on emotional prosody identification in paranoid schizophrenia. Psychol Med. 2008/11/13. 2009;39: 927–938. doi:10.1017/S0033291708004704
3. Haas EC, Casali JG. Perceived urgency of and response time to multi-tone and frequency-modulated warning signals in broadband noise. Ergonomics. 1995;38: 2313–2326. doi:10.1080/00140139508925270
4. Suied C, Susini P, McAdams S. Evaluating Warning Sound Urgency With Reaction Times. J Exp Psychol Appl. 2008;14: 201–212. doi:10.1037/1076-898X.14.3.201
5. Arnal LH, Flinker A, Kleinschmidt A, Giraud AL, Poeppel D. Human Screams Occupy a Privileged Niche in the Communication Soundscape. Curr Biol. 2015;25: 2051–2056. doi:10.1016/j.cub.2015.06.043
6. Engelberg JWM, Gouzoules H. The credibility of acted screams: Implications for emotional communication research. Q J Exp Psychol. 2019;72: 1889–1902. doi:10.1177/1747021818816307
7. Arnal LH, Flinker A, Kleinschmidt A, Giraud AL, Poeppel D. Human Screams Occupy a Privileged Niche in the Communication Soundscape. Curr Biol. 2015;25: 2051–2056. doi:10.1016/j.cub.2015.06.043
8. Trevor C, Arnal LH, Frühholz S. Terrifying film music mimics alarming acoustic feature of human screams. J Acoust Soc Am. 2020;147: EL540-EL545. doi:10.1121/10.0001459
9. Grandjean D, Sander D, Pourtois G, Schwartz S, Seghier ML, Scherer KR, et al. The voices of wrath: Brain responses to angry prosody in meaningless speech. Nat Neurosci. 2005/01/25. 2005;8: 145–146. doi:10.1038/nn1392
10. Frühholz S, Hofstetter C, Cristinzio C, Saj A, Seeck M, Vuilleumier P, et al. Asymmetrical effects of

unilateral right or left amygdala damage on auditory cortical processing of vocal emotions. Proc Natl Acad Sci U S A. 2015;112: 1583–1588. doi:10.1073/pnas.1411315112
11. Lavan N, Burston LFK, Ladwa P, Merriman SE, Knight S, McGettigan C. Breaking voice identity perception: Expressive voices are more confusable for listeners. Q J Exp Psychol. 2019;72: 2240–2248. doi:10.1177/1747021819836890
12. Eisenberger NI. The neural bases of social pain: Evidence for shared representations with physical pain. Psychosom Med. 2012;74: 126–135. doi:10.1097/PSY.0b013e3182464dd1
13. Bach DR, Dolan RJ. Knowing how much you don't know: A neural organization of uncertainty estimates. Nature Reviews Neuroscience. 2012. pp. 572–586. doi:10.1038/nrn3289
14. Hernandez-Lallement J, Attah AT, Soyman E, Pinhal CM, Gazzola V, Keysers C. Harm to Others Acts as a Negative Reinforcer in Rats. Curr Biol. 2020;30: 949–961.e7. doi:10.1016/j.cub.2020.01.017
15. Sauter DA, Eisner F, Calder AJ, Scott SK. Perceptual cues in nonverbal vocal expressions of emotion. Q J Exp Psychol. 2010/05/04. 2010;63: 2251–2272. doi:10.1080/17470211003721642
16. Schwartz JW, Engelberg JW, Gouzoules H. What is a scream? Acoustic characteristics of a human call type. J Acoust Soc Am. 2019;145: 1776–1776. doi:10.1121/1.5101500
17. Anikin A, Bååth R, Persson T. Human Non-linguistic Vocal Repertoire: Call Types and Their Meaning. Journal of Nonverbal Behavior. 2018: 53–80. doi:10.1007/s10919-017-0267-y
18. Gouzoules H, Gouzoules S, Tomaszycki M. Agonistic screams and the classification of dominance relationships: Are monkeys fuzzy logicians? Anim Behav. 1998;55: 51–60. doi:10.1006/anbe.1997.0583
19. Gouzoules H, Gouzoules S. Agonistic screams differ among four species of macaques: The significance of motivation-structural rules. Anim Behav. 2000;59: 501–512. doi:10.1006/anbe.1999.1318
20. Mercier S, Déaux EC, van de Waal E, Bono AEJ, Zuberbühler K. Correlates of social role and conflict severity in wild vervet monkey agonistic screams. PLoS One. 2019;14. doi:10.1371/journal.pone.0214640
21. Belin P, Zatorre RJ. Neurobiology: Sounding the Alarm. Current Biology. 2015. pp. R805–R806. doi:10.1016/j.cub.2015.07.027
