# Supplementary material for: Neurocognitive processing efficiency for discriminating human non-alarm rather than alarm scream calls
Source: PLoS Biol. 2021 Apr 13;19(4):e3000751. doi: 10.1371/journal.pbio.3000751 (PMC8043411; doi:10.1371/journal.pbio.3000751)
Supplement: Attachment — Submitted filename: SFruhholz_reponse_to_reviewers.pdf [file pbio.3000751.s016.pdf]

Reponses to comments by the reviewers
PBIOLOGY-D-20-01112R2

REVIEWERS' COMMENTS:
(these should be the correct numbering, as per the original version; apologies for the previous reviewer numbering error)

Reviewer #1:

I feel that the authors have made a nice job in answering the reviewers' suggestions and I think them for their effort. I am satisfied with the results from the replication experiment that suggests that stimulus selection does not represent a relevant bias. Also the non-significant effect of original loudness of stimuli suggests that the observed effects are not primarily driven by the standardization procedure.

Response: We thank the reviewer for this positive evaluation of our second revised manuscript. We have taken on board the final two comments by the reviewer as mentioned below.

The current manuscript is much more balanced in the sense that the authors acknowledge the importance of the task under investigation (which may also party explain the relative strong fMRI response to neutral stimuli during the gender discrimination task - an interpretation that should be added to the manuscript).

Response: This notion was now included in the manuscript, p21:

"This gender discrimination task might have also led to a relatively strong neural activity for neutral scrams in comparison to some other types of screams especially of an alarming nature. However, previous studies using a gender discrimination task during the processing of vocal emotions usually found higher activity for negative compared to neutral vocal emotions, and the implicit processing of the affective quality of voice signals has been demonstrated by many previous studies [1,2]."

The only two parts of the manuscript that still allow for misinterpretation are the title and the abstract. I believe that "Neurocognitive processing efficiency for discriminating human non-alarm rather than alarm scream calls" would not diminish the significance of the paper in any way and more specifically describe what the paper is mainly about.

Response: We changed the title according to the reviewer's suggestion.

In the same line, the last sentence of the abstract should highlight what the authors can indeed claim, for example: ...in humans and that any potential threat processing bias does not translate into a higher efficiency during scream discrimination or into increased implicit processing when identifying the screaming person's gender."

Response: We changed the last sentence of the abstract to better reflect the conclusions on our data. We would however, like to note that the suggestion made by the reviewer might sound a little bit ambiguous. Especially for the part "…and that any potential threat processing bias does not translate into a higher efficiency during scream discrimination"; we feel that this reads rather ambiguous and would actually suggest that we did not find any difference between the behavioral and neural processing of different scream types (i.e. if a bias, be it negative or positive, does not translate into category discrimination, than no differences should be expected), and we think this is therefore not the best summary of our data, since we indeed found differences in the processing of different types of screams. To recur to the notion of threat processing bias as suggested by the reviewer, and to specifically describe the task-context of our significant findings, we changed the last sentence as follows, p2:

"These findings show that scream calls are more diverse in their signaling and communicative nature in humans and, unlike a commonly observed threat processing bias in perceptual discriminations and neural

processes, we found that especially non-alarming screams, and positive screams in particular, seem to have higher efficiency in speeded discriminations and the implicit neural processing of various scream types in humans".

Reviewer #2:

The authors present a good revision of their manuscript. They are now much more cautious with how they interpret their results, and integrated some alternative explanations for these results in the discussion. I am happy with how they dealt with my comments and have no further remarks.

Response: We thank reviewer #2 for this final and very positive evaluation of our manuscript.

Reviewer #4:
[identifies himself as Harold Gouzoules]

Frühholz et al. have made brief changes in this revision that are consistent with, and largely responsive to, my comments on the previous draft. Ideally, additional fleshing out of some of the points would have been desirable, but because the article is to be of the "short report" form, I won't ask for additional changes other than a couple of points noted below.

Response: We thank the reviewer for this positive feedback on our manuscript.

The authors have very nicely responded to my request for a more accurate account of the nonhuman primate literature on the use of screams during agonistic encounters. One very slight change would enhance the account. In the revision, they say: "Screams by lower-ranking animals help to recruit support from allies [18,19], while higher-ranking animals scream to intimidate the lower-ranking animal when challenged by this lower-ranking opponent [20]".

The use of screams by dominant monkeys when challenged by a lower-ranking individual is not a matter of intimidation. Instead, those screams also recruit support from matrilineal kin … just as screams do for subordinate monkeys attacked by a dominant individual. The difference is that a monkey will use an acoustically different scream when confronted by a dominant opponent compared to when it is challenged by a subordinate individual. The different types of screams thus provide listeners with information about the nature of the fight, and the kind of response (intervention and assistance) they show is influenced by the scream type they hear.

Response: The respective sentence on was changed accordingly, p3:

"Screams by lower-ranking animals help to recruit support from allies [3,4], while higher-ranking animals scream to recruit support from matrilineal kin when challenged by lower-ranking opponents [5]."

Although I have not previously "weighed in" on the other reviewers' comments or Frühholz et al.'s responses to them, it seems to me that, in the case below, the matter debated could be resolved by the proposal that the recognition accuracy question makes more sense from an evolutionary cost-benefit perspective. The issue:

Reviewer 2 says: " ….. It seems clear to me, that, based on the results presented by the authors, non-alarm screams have a higher discriminability advantage compared to alarm screams among themselves (among these three categories). This however, does not mean that alarm screams have a disadvantage. …."

Frühholz et al. respond: "Furthermore, the observation that alarm screams categories are often used during misclassification of all screams ('alarm scream categories have some primacy during misclassification of other scream types') can be a topic of debate. If alarm scream categories are often used during misclassification of any type of scream, this again does not speak for a processing "advantage". A cognitive system usually wants to achieve high recognition accuracy, so introducing a factor in the recognition process that facilitates classification errors is surely not of "advantage" for a human recognition system. So, we are unsure what the reviewer means by stating that this 'seems contradictory with the claim that the authors are trying to make'."

I suggest that "errors" or perceptual bias in a communication system might be advantageous from an evolutionary perspective, one that goes beyond a view rooted exclusively in cognitive efficiency and accuracy. Specifically, given the evolutionary origins of screams (calls given by prey in the grasp of a predator) and the associated emotional state of fear, processing errors that bias "non-alarm screams" toward "alarm screams" likely have less cost than the reverse error (i.e., failing to process a true "alarm scream" as such). The adaptive response or "bias" is the one where the errors have less cost, and misclassifying a non-alarm scream as an alarming one is likely less harmful than perceiving an alarm scream as non-alarm. I think the "advantage - disadvantage" disagreement above is thus misguided. Frühholz et al.'s persistence with the interpretation that alarm screams are disadvantaged is the main source of confusion here, I think, and along with reviewer 2, I consider the conclusion problematic.

Response: The reviewer #4 mentions an important point here that was a matter of discussion and clarification with reviewer #2 during the two first rounds of revisions. In agreement with the comments of reviewer #2, we edited our manuscript along with the suggestions made by this reviewer and included a more detailed discussion on this topic, especially in the second round of the revision process. Reviewer #2 now seems satisfied with the changes and edits that we included in the second revised version of the manuscript, and we think this now gives a clear and detailed interpretation of our data, with alternative explanations now being extensively discussed in the manuscript.

We agree with the reviewer that the advantage-disadvantage issue (i.e. making errors is in principle a disadvantage) could be extended by the discussion about the cost-benefits of making decisional errors in scream classifications (i.e. reducing the likelihood of “miss” decisions on potential alarm and threat). We however feel that this extended discussion as suggested by reviewer #4 here was already explicitly included in the previous second revised manuscript, where we wrote on p18:

“This might resemble a natural threat perception bias that seems to be a cost-benefit efficient solution when balancing response options against potential sources of (non-)threat, especially under conditions of uncertainty [6]. Although perceptual misclassifications seem to be of general disadvantage to any recognizing organism, these misclassifications as “alarm” screams might figure as a safer option in potential threat recognition.”

In the third revised manuscript, we now extended this discussion a little bit to make this point even more explicit and clearer, p18-19:

“This might resemble a natural threat perception bias that seems to be a cost-benefit efficient solution when balancing response options against potential sources of (non-)threat, especially under conditions of uncertainty [6]. Although perceptual misclassifications seem to be of general disadvantage to any recognizing organism, these misclassifications as “alarm” screams might figure as a safer option in potential threat recognition. This might also be rooted in the evolutionary primacy of alarm screams to signal threat and elicit fear in receivers, and which required immediate adaptive response in the organisms targeted by the threat. Threat avoidance is thus the primary concern of an organism, and a ”bias” towards incorrectly classifying non-alarm screams as threatening alarms is safer for an organism than performing the reverse error.”

References

1. Grandjean D, Sander D, Pourtois G, Schwartz S, Seghier ML, Scherer KR, et al. The voices of wrath: Brain responses to angry prosody in meaningless speech. Nat Neurosci. 2005/01/25. 2005;8: 145–146. doi:10.1038/nn1392
2. Frühholz S, Hofstetter C, Cristinzio C, Saj A, Seeck M, Vuilleumier P, et al. Asymmetrical effects of unilateral right or left amygdala damage on auditory cortical processing of vocal emotions. Proc Natl Acad Sci U S A. 2015;112: 1583–1588. doi:10.1073/pnas.1411315112
3. Gouzoules H, Gouzoules S, Tomaszycki M. Agonistic screams and the classification of dominance relationships: Are monkeys fuzzy logicians? Anim Behav. 1998;55: 51–60. doi:10.1006/anbe.1997.0583
4. Gouzoules H, Gouzoules S. Agonistic screams differ among four species of macaques: The significance of motivation-structural rules. Anim Behav. 2000;59: 501–512. doi:10.1006/anbe.1999.1318
5. Mercier S, Déaux EC, van de Waal E, Bono AEJ, Zuberbühler K. Correlates of social role and conflict severity in wild vervet monkey agonistic screams. PLoS One. 2019;14. doi:10.1371/journal.pone.0214640
6. Bach DR, Dolan RJ. Knowing how much you don’t know: A neural organization of uncertainty estimates. Nature Reviews Neuroscience. 2012. pp. 572–586. doi:10.1038/nrn3289
